# AI-Driven Spectral Decomposition: Predicting the Most Probable Protein Compositions from Surface Enhanced Raman Spectroscopy Spectra of Amino Acids

**DOI:** 10.3390/bioengineering11050482

**Published:** 2024-05-12

**Authors:** Siddharth Srivastava, Nehmat Sandhu, Jun Liu, Ya-Hong Xie

**Affiliations:** Department of Materials Science and Engineering, University of California, Los Angeles, CA 90095, USA; sidsri@g.ucla.edu (S.S.); nehmatsandhu@g.ucla.edu (N.S.); ljun@ucla.edu (J.L.)

**Keywords:** SERS, biosensing, neural networks, proteomics, plasmonics, Raman spectroscopy

## Abstract

Surface-enhanced Raman spectroscopy (SERS) is a powerful tool for elucidating the molecular makeup of materials. It possesses the unique characteristics of single-molecule sensitivity and extremely high specificity. However, the true potential of SERS, particularly in capturing the biochemical content of particles, remains underexplored. In this study, we harnessed transformer neural networks to interpret SERS spectra, aiming to discern the amino acid profiles within proteins. By training the network on the SERS profiles of 20 amino acids of human proteins, we explore the feasibility of predicting the predominant proteins within the µL-scale detection volume of SERS. Our results highlight a consistent alignment between the model’s predictions and the protein’s known amino acid compositions, deepening our understanding of the inherent information contained within SERS spectra. For instance, the model achieved low root mean square error (RMSE) scores and minimal deviation in the prediction of amino acid compositions for proteins such as Bovine Serum Albumin (BSA), ACE2 protein, and CD63 antigen. This novel methodology offers a robust avenue not only for protein analytics but also sets a precedent for the broader realm of spectral analyses across diverse material categories. It represents a solid step forward to establishing SERS-based proteomics.

## 1. Introduction

Surface-enhanced Raman spectroscopy (SERS) plays a pivotal role in molecular studies, particularly in analyzing amino acids and proteins [1]. Its capability to intensify the highly specific Raman signals from molecules on rough metal surfaces like gold or silver has made it widely applicable across various disciplines [2]. This specificity arises from the inelastic scattering of light, where photons interact with molecular vibrations, producing a distinct spectral fingerprint unique to each molecule [3]. SERS’s unique ability for high sensitivity and specificity in biomolecular fingerprinting is notable. It enables detailed exploration of molecular structures with exceptional precision, especially in differentiating subtle molecular compositions [2] and even secondary configurations [4,5]. This ability to distinguish minute differences in molecular compositions and configuration makes it an invaluable tool in the study of biomolecules, unveiling layers of complexity that were previously inaccessible, and in a non-destructive manner. The intricate detail SERS offers in analyzing complex molecules like amino acids and proteins, which are fundamental to life, is remarkable [1,6].

Understanding amino acid composition in proteins is critical in advancing fields like biotechnology and medical research [7]. Amino acids, the building blocks of proteins, define their structure, impact their functionality, and affect their interactions within biological systems. This comprehension is essential for protein identification, and understanding structural complexities, and functionalities [8,9,10].

In medical research, analyzing amino acid composition in proteins is key to understanding the molecular basis of diseases. It is not only the amino acid composition of proteins, but their secondary folding configuration also that plays a very important role in their biological functionalities [4,11]. For example, in hemoglobin, changes in amino acid composition and structure can lead to disorders like sickle cell anemia [12]. The amino acid composition also provides insights into disease mechanisms, leading to targeted therapies with the potential for greater efficacy and fewer side effects [13,14]. In biotechnology and food science, protein engineering—altering amino acid sequences—enables the creation of proteins with specific properties, crucial in industrial processes, food production, and pharmaceutical development. For instance, proteins designed for thermostability can enhance efficiency in various applications. Additionally, in antimicrobial peptides (AMPs), amino acid composition is critical for effectiveness, and in food science, it is vital for nutritional assessment and development [14,15,16,17]. Furthermore, in view of SERS being a non-destructive method that possesses sensitivity to the secondary structure but also has demonstrated capability in rendering detailed conformational information, as illustrated in our study of Aβ40 and Aβ42 [4,18], it in principle could be a powerful proteomics tool.

Despite its importance, current methods for determining amino acid composition, essentially proteomic methods, face challenges. Techniques like amino acid analysis (AAA) [19] and mass spectrometry (MS) [20], are some of the prominent techniques. While MS, in its various forms including tandem MS, ion mobility, and high-resolution MS, represents a highly powerful methodology for the detection, identification, and quantification of proteins, peptides, and amino acids, challenges persist in certain contexts. These techniques may require extensive sample preparation, and processing time, and often do not provide direct information about the protein’s functional state or its interactions within a cellular context. These methods often necessitate the destruction of the sample, which can be a significant limitation. The requirement for hydrolysis (in AAA) or ionization (in MS) can lead to the loss of spatial information and potentially alter the native state of proteins or peptides. Furthermore, both AAA and MS demand specialized equipment, technical expertise, and often costly reagents, making them less accessible for rapid or field-based applications. Hence, despite the undeniable capabilities of AAA and MS, there is a pressing need for alternative, or even complementary, technologies that provide a rapid, non-destructive, direct, and cost-effective method to determine amino acid composition. This study introduces a novel [20,21,22,23] approach by leveraging the power of Surface-Enhanced Raman Spectroscopy (SERS) combined with transformer neural networks. Table 1 gives an overview of MS, AAA, and SERS in proteomic research, where we mention the opportunities and limitations for each.

But first, there is a need to acknowledge certain limitations of SERS itself, which highlight the need for the present study. SERS signal strength can vary significantly with slight changes in the experimental setup, including substrate composition, laser wavelength, and sample preparation, making reproducibility a challenge [24,25]. The richness of SERS spectra, while a potential advantage, also poses a challenge for extracting specific proteomic information due to overlapping signals from complex biological mixtures. Additionally, unlike MS, where extensive databases exist for peptide and protein identification, SERS lacks a comparable, comprehensive spectral library for proteins and peptides, hindering its ability to identify and quantify them directly from SERS spectra. All these limitations combined, represent the difficulty and uncertainties in realizing SERS-based proteomics.

Much of these limitations can be addressed, to some extent, by utilizing machine learning-based data analysis. Historically, the study of complex biomolecules using SERS was challenging due to the subtle nuances in their spectral signatures. These nuances, often representing minute conformational changes or structural variations, are of paramount importance, especially when decoding biological processes or discerning between molecular isoforms [3]. Modern SERS instruments with high-resolution capabilities produce complex spectra, demanding sophisticated analytical techniques. Machine learning shows promise in handling these complex datasets, especially relevant being the ability to identify and “learn” interrelation between complex features in the data [26]. Hence, the synergy of SERS and machine learning forms the core of our study. 

The rapid advancement of spectroscopic methods has generated data of increasing quantity and complexity, which has brought forth a pressing need for equally sophisticated analytical techniques. Machine learning, with its promise of discerning patterns in large datasets, has shown immense potential in image recognition, natural language processing, and predictive analytics [27]. Within this domain, a newer paradigm, transformer neural networks, originally designed to handle sequences in language processing tasks, has shown outstanding promise [28]. We explore whether this architecture, which has revolutionized natural language processing, holds the key to decoding the rich tapestries of SERS spectra. 

By harnessing the analytical prowess of transformer neural networks, we aspire to address the challenge of identifying hidden intricacies within the spectra, fostering a novel approach to proteomic analysis that is both sophisticated and efficient. Originally designed to manage sequential data in natural language processing tasks, transformers brought the concept of “attention”—allowing the model to focus on different parts of the input data differently, akin to how humans pay attention to specific words or phrases when understanding a sentence [28]. Similarly, we could utilize this mechanism to identify the important features in specific SERS spectra presented to the model. 

Our goal was to leverage the synergistic potential of SERS and artificial intelligence to revolutionize our understanding of protein compositions and functions. Firstly, we aimed to leverage the prowess of transformer neural networks to aid in spectral analysis. Given the sequential nature of spectra, where each data point (wavenumber) has a relationship with its neighbors, transformers, with their attention mechanisms, seemed a natural fit [29]. Our second objective was to showcase how AI-driven techniques, particularly when decoding complex SERS spectra, could lead the way toward methodologies that are non-destructive and non-invasive, ensuring the integrity and sanctity of samples. 

Our methodology promises to make positive strides in the analysis of amino acid composition in proteins, offering a swift, direct, and non-invasive alternative to traditional techniques. The implications of this research promise significant advancements in protein analytics and disease understanding. 

**Table 1:** Comparative overview of SERS, mass spectrometry, and amino acid analysis for proteomic research.

Aspect	SERS [30,31] + ML	Mass Spectrometry (MS) [20,23]	Amino Acid Analysis (AAA) [19,32]
Method Overview	Utilizes Raman scattering to identify molecules based on their vibrational modes. Enhanced by metallic nanostructures for increased sensitivity.	Separates and identifies compounds based on their mass-to-charge ratio. Can be coupled with chromatography for complex mixtures.	Relies on the separation and quantification of free amino acids, typically involving pre-column derivatization followed by HPLC.
Specificity	High specificity, with molecular fingerprints providing unique spectral signatures.	High, especially when combined with tandem MS for structural analysis.	High for individual amino acids but does not provide information on sequence or modifications.
Sample Preparation	Minimal. Direct analysis possible with little to no sample preparation for certain samples.	Often requires extensive sample preparation, including purification and concentration steps.	Requires hydrolysis (which can lead to destruction or loss of certain amino acids), followed by derivatization.
Analysis Time	Rapid, with spectra acquisition in seconds to minutes per sample.	Can vary from minutes to hours, depending on the complexity of the sample and the setup.	Lengthy, with hydrolysis taking several hours to complete, followed by the derivatization and separation process.
Limitations	Sensitivity and reproducibility can be influenced by the substrate and experimental setup.	High operational costs and complex sample preparation. Requires expert operation and maintenance.	Destruction of the sample through hydrolysis. Does not provide information on protein folding or conformation.
Potential for Proteomics	Demonstrated potential for amino acid and protein composition analysis. May require further development for complex proteomic applications.	Widely used in proteomics for identifying and quantifying proteins and peptides, including post-translational modifications.	Primarily used for quantitative amino acid analysis; limited utility in proteomics without additional techniques for protein identification.

## 2. Materials and Methods

### 2.1. SERS Substrate Fabrication

The SERS substrate utilized in this study is based on our previously developed platform, detailed in references [33,34,35,36]. Our “gold nanopyramid platform” is a thin film substrate of gold (Au) with an array of pyramidal nanostructures. We have provided the scanning electron microscopy (SEM) images of our Au Nanopyramid SERS Substrate in Section S3 of the supplementary file. The fabrication steps were as follows:

- Formation of Polystyrene Ball Layer: A monolayer of polystyrene (PS) balls, each 500 nm in diameter, was formed on a water surface through Langmuir–Blodgett patterning for self-assembly.
- Transfer and Deposition: This PS ball layer was transferred onto a 4-inch (001) silicon wafer pre-coated with a 50 nm layer of SiO_2_, followed by the sputter deposition of a 50 nm chromium (Cr) layer.
- PS Ball Removal: The PS balls were removed using chloroform, exposing the SiO_2_ layer, which was then etched using reactive-ion etching to reveal the desired pattern.
- Silicon Etching: The silicon substrate was etched with KOH, creating inverted nanopyramids with 57.5° angle sidewalls by exploiting the differential etching rates of the [001] and [111] crystal directions.
- Gold Film Deposition: Finally, a 200 nm thick gold film was deposited onto the etched surface using electron beam deposition, and the substrate was bonded to a carrier wafer with epoxy before the gold film was lifted off.

### 2.2. Raman Spectroscopy 

The Raman mapping parameters were tailored to suit the specific requirements of this study:

- Sample Preparation: 5 μL of each sample solution was deposited on the SERS substrate and dried before Raman testing.
- Spectrometer: Measurements were performed using a Renishaw inVia Raman spectrometer at room temperature, with a laser excitation wavelength of 785 nm and a power of 5 mW.
- Calibration: The system was calibrated using the 520 cm^−1^ peak of silicon.
- Coarse Mapping: This is to first identify the locations on the substrate with high signal density from the analyte, an initial scouting for particle locations was performed at a step width of 2 μm, with an exposure time of 0.2 s to prevent sample overheating.
- Fine Mapping: After spotting particles, fine mapping was performed at a step width of 0.1 μm to collect characteristic spectra from the sample, maintaining the exposure time of 0.2 s to avoid overheating. This allowed for a more detailed spectral map of the locations identified in the previous step, with a higher signal quality, which were subsequently used to train ML models.

**Figure 1:**
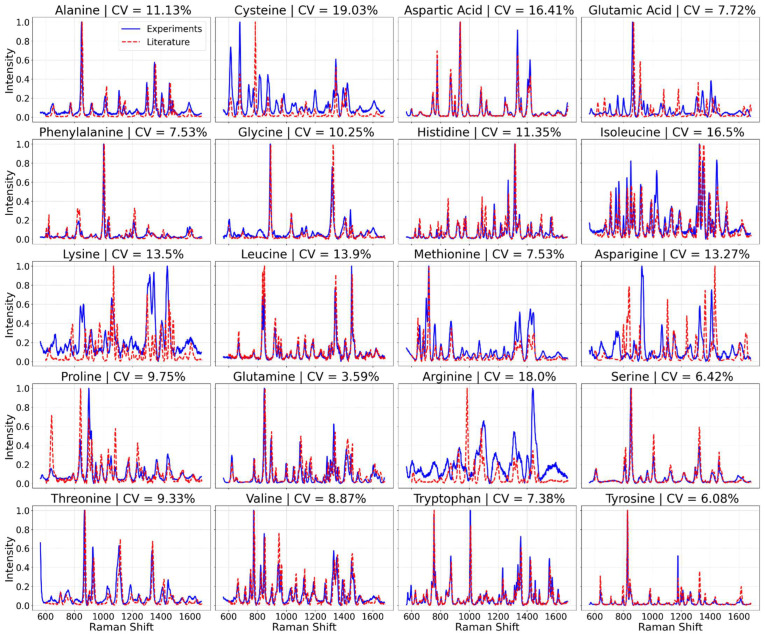
Averaged SERS spectra of the 20 amino acids.

Additionally, to compare our experimental results with previous data and to showcase both the internal spectral consistency within a fixed experimental setup and the spectral variance due to changes in experimental conditions, we overlaid amino acid spectra obtained from previous studies [37,38] with our own spectra. These comparisons are presented in Figure 1.

The SERS data collection was carried out in 2 batches for each amino acid—first, to obtain dataset to train and test the machine learning models; second, to obtain an independent dataset to validate the model performance. The data are presented in Section S2 of the supplementary file.

### 2.3. Data Collection and Preprocessing 

Our study began with the systematic collection of SERS spectra from 20 amino acids, each prepared in a 0.2 milli-molar (mM) solution using deionized (DI) water to ensure uniformity and consistency in the spectral analysis. The use of DI water as a solvent was crucial for maintaining the purity and integrity of each solution, minimizing potential interference from impurities. To address spectral signature fluctuations caused by sample variations, SERS platform heterogeneity, and instrument fluctuations, we applied specific preprocessing techniques. This included fluorescence background subtraction using asymmetric least square fitting and noise reduction via Savitzky–Golay filtering, both conducted through batch processing to effectively isolate the Raman signal from the fluorescence component and smooth the spectra while preserving critical features. Subsequent min–max normalization proportionally compressed the original intensity range to a [0, 1] scale, harmonizing the data scale. This normalization, alongside other preprocessing methods like wavelet-based methodologies for signal-to-noise ratio enhancement and baseline correction for background noise elimination [39,40,41], was essential in ensuring the spectra’s quality and suitability for analysis with our sophisticated machine learning models. Furthermore, since the amino acids do not contain peptide bonds, but the proteins under consideration do, the SERS peaks corresponding to peptide bonds [11] were removed for the purpose of analysis, to ensure biochemical accuracy in model training and application. 

Furthermore, given the initial collection of 2420 spectra, with each of the 20 amino acids contributing 121 spectra obtained via Raman mapping, the variability in signal quality was a significant concern. To address this, we employed a signal-to-noise ratio (SNR) based sorting method as a criterion for data quality assessment and filtration.

Signal-to-Noise Ratio Assessment and Thresholding: Each spectrum’s SNR was calculated and assessed to identify and exclude data compromised by excessive noise. Through a combination of trial and error and visual analysis of the spectra, an SNR threshold of 50 was established as the optimal balance between data quality and quantity. Spectra falling below this threshold were considered unsuitable for reliable analysis due to their diminished signal clarity and were thus excluded from the dataset.

Selection of High-Quality Spectra: Post-SNR assessment, the top 33 spectra for each amino acid, meeting or exceeding the SNR threshold, were selected for further analysis. This decision was informed by the need to maintain a uniform number of spectra per amino acid to avoid potential data imbalance that could skew the clustering results. This uniformity ensures that the subsequent analysis is not biased towards amino acids with initially higher numbers of high-SNR spectra.

### 2.4. Clustering Analysis Using t-SNE

In our study, we conducted a comprehensive clustering analysis to examine the intrinsic groupings within the high-dimensional amino acid spectra, where ‘high-dimensional’ refers to the 1117 Raman shifts per spectrum. We utilized t-Distributed Stochastic Neighbor Embedding (t-SNE), a machine learning algorithm for dimensionality reduction well-suited for visualizing high-dimensional datasets. The aim was to reduce the dimensions to a 3D space that preserves the local structure and relationships inherent in the amino acid spectral data, facilitating an understanding of the similarities and distinctions among the different amino acids. The dimensionality reduction through t-SNE transformed the 1117-dimensional space into a 3-dimensional representation, which enabled a more comprehensive view of the data structure for subsequent analysis. The open-source scikit-learn library in Python was utilized for this purpose.

### 2.5. Neural Network Model Architecture for Classification 

We employed the transformer architecture, a model adept at processing sequential data, originally from natural language processing [28]. Its self-attention mechanism is well-suited for identifying patterns in SERS spectra. The model includes multiple encoder layers with multi-head self-attention mechanisms and feed-forward neural networks, enhanced by layer normalization and residual connections for stability [42]. The model architecture utilized is presented in Section S1 of the supplementary information file.

### 2.6. Training Procedure 

Our training process was designed to address a dataset comprising 660 amino acid spectra, each characterized by 1117 Raman shifts. We employed the Adam optimizer, known for its efficiency in handling large-scale datasets [43]. The optimizer started with an initial learning rate of 0.001, and we adopted a dynamic approach to adjust this rate during training for optimal model performance. In order to mitigate the risk of overfitting, a dropout rate of 0.3 was applied after each dense layer. This strategy maintained a balance between the model’s complexity and its generalization ability, which is critical for predictive accuracy.

The dataset was segmented into training (80%) and test (20%) sets. This dataset division was meticulously performed using the “train_test_split” function from the scikit-learn library in Python. This method ensures random shuffling of the data prior to the split, promoting a fair and unbiased distribution of samples across both datasets. The chosen 80–20 split optimally balances the need for a substantial amount of data for training the model, ensuring it learns the complex patterns within the SERS spectra, while still reserving enough unique samples to rigorously evaluate the model’s predictive accuracy on unseen data. This distribution was crucial for robust training, allowing for precise tuning of hyperparameters during validation and providing a comprehensive evaluation of the model on unseen data. The test set consisted of protein spectra, which differed from the amino acid spectra used in training, thereby testing the model’s predictive capabilities in a distinct context.

The model was configured for a 20-class classification task, reflecting the 20 standard amino acids. The output layer of the model produced 20 probabilities, each corresponding to one of the 20 amino acids. This design enabled the model to predict the likelihood of each amino acid’s presence in the given spectra. Throughout the training, early stopping and model checkpointing were implemented as callbacks. Early stopping monitored the validation loss with patience of five epochs, halting the training at the most effective point to prevent overfitting. Model checkpointing ensured the retention of the best model version based on the minimum validation loss.

Post-training, the model was utilized to predict amino acid compositions in protein spectra from the test set. These predictions were then compared with actual amino acid compositions from the Uniprot [44] database of proteins, which contains peer-reviewed data on amino acid sequences of proteins. This comparative analysis was pivotal in evaluating the model’s accuracy and practical utility in deducing protein structures and functions based on their constituent amino acids.

The machine learning approach offers significant strengths, such as the ability to process complex, high-dimensional data, and provide advanced analysis and prediction capabilities beyond traditional methods. This is particularly beneficial in spectral analysis for obtaining detailed insights into molecular structures, where identifying patterns and meaning behind the data is non-trivial. The utilization of dynamic learning rate adjustments and dropout strategies enhances the model’s generalization across datasets. However, challenges in interpretability arise from the opaque nature of deep learning, limiting the clarity of insights derived from the model, especially in intricate biochemical contexts. Additionally, our model’s effectiveness is heavily dependent on the quality and diversity of the training data, with limited or biased datasets potentially undermining accuracy and reliability. 

### 2.7. Calculating Coefficient of Variation

To quantitatively calculate reproducibility from your spectral data, a commonly used metric is the coefficient of variation (CV), which is the ratio of the standard deviation (σ) to the mean (μ) of a dataset, expressed as a percentage:

$$CV=(\frac{\sigma }{\mu })\times 100\%$$

The CV provides a standardized measure of dispersion of a probability distribution or frequency distribution. In the context of SERS spectra, it can be applied to the intensities of specific Raman peaks across multiple spectra to assess their reproducibility [45,46].

This threshold is often considered acceptable in analytical chemistry and bioanalytical assays, indicating that the data are sufficiently consistent across multiple measurements. However, the acceptability of a specific CV value can depend on the context of the experiment and the inherent variability of the method being used. In cases where extremely precise measurements are required, a lower CV threshold might be more appropriate. 

### 2.8. Error Metrics 

In the evaluation of our machine learning model’s performance, two critical statistical measures were employed: the root mean square error (RMSE) and the mean squared error (MSE) [47]. These metrics are paramount for assessing the accuracy of the model’s predictions in relation to the actual amino acid compositions obtained from the SERS spectra.

The mean squared error (MSE) is defined as the average of the squared differences between the predicted values (${\hat{y}}_{i}$) and the actual values ($y_{i}$). Mathematically, it is represented as: $MSE=\frac{1}{n}\sum_{i=1}^{n}{\left({\hat{y}}_{i}-y_{i}\right)}^{2}$, where *n* is the number of observations. The squaring of the errors ensures that larger errors are penalized more heavily, making MSE a robust measure of model performance, particularly in highlighting significant deviations between predicted and observed values.

The root mean square error (RMSE) further builds on the MSE by taking its square root, thus bringing the error metrics back to the original units of the data, facilitating easier interpretation: $RMSE=\sqrt{\frac{1}{n}\sum_{i=1}^{n}{\left({\hat{y}}_{i}-y_{i}\right)}^{2}}$. RMSE offers a straightforward measure of the average magnitude of the model’s prediction errors, providing insights into how accurately the model can predict the amino acid compositions from SERS spectra.

## 3. Results

### 3.1. SERS Spectra of Amino Acids

Our exploration of the surface-enhanced Raman spectroscopy (SERS) spectra of 20 amino acids revealed a comparison between the averaged spectra from our laboratory experiments and those reported in the existing literature, as shown in Figure 1. The spectra we obtained, illustrated in blue, exhibited discrepancies when compared with previous studies’ spectra, shown in red.

We present averaged SERS spectra for each amino acid, based on 75 individual spectra per amino acid. Notable observations include tryptophan’s strong Raman bands around 1552 cm^−1^ and 760 cm^−1^, alanine’s peak around 1445 cm^−1^, and phenylalanine’s bands at approximately 1000 cm^−1^ and 1033 cm^−1^.

The coefficient of variation (CV) for the ten major Raman bands’ peak intensities across measurements for each amino acid was calculated. While most amino acids showed CV values below 10%, indicating high reproducibility [37,38,48,49].

### 3.2. Clustering of Amino Acid Spectra

**Figure 2:**
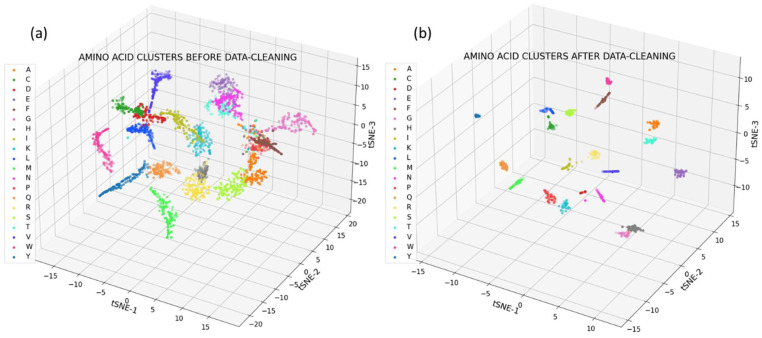
Unsupervised clustering of the amino acid dataset (**a**) before filtering, and (**b**) after filtering by signal-to-noise ratio.

Through the utilization of t-SNE for clustering analysis, our study has identified 20 distinct clusters from a dataset of 2420 SERS spectra, encompassing 121 spectra from each of the 20 amino acids. These clusters, depicted in Figure 2a, illuminate the inherent groupings within our spectral data, offering a visual representation of the amino acids’ distinctiveness.

Further refinement was achieved by enhancing data quality through SNR-based selection, narrowing down to the top 33 spectra for each amino acid. This refined clustering, visible in Figure 2b, resulted in clearer and more distinct cluster formations. This process underlines the significant role of data quality in the discernment of amino acid spectra.

### 3.3. Model Training and Performance on Amino Acids 

**Figure 3:**
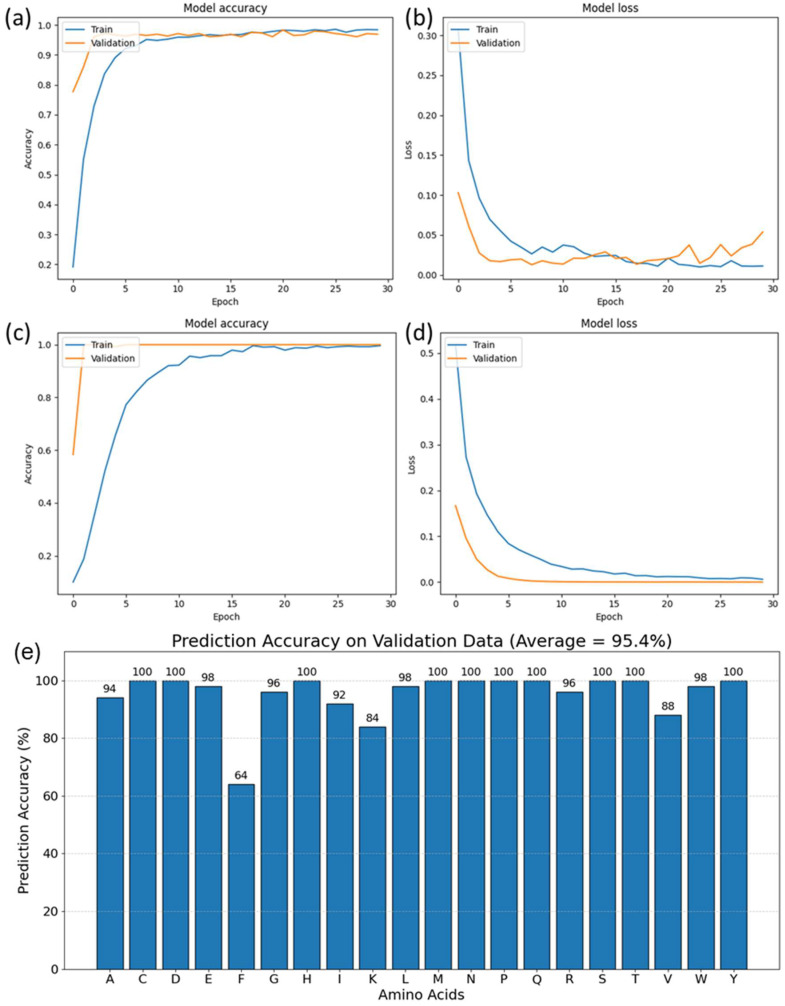
Model training performance on the amino acid data. (**a**,**b**) Accuracy and loss curves for training on unfiltered dataset, (**c**,**d**) accuracy and loss curves for training on filtered dataset, (**e**) prediction accuracy on validation dataset of amino acids.

Our investigation utilized t-SNE clustering to illustrate the distinctness between amino acid datasets, which served as a precursor for more detailed analysis. The subsequent step involved training a transformer neural network model capable of predicting amino acid compositions from SERS spectra. The training process and its outcomes are depicted in Figure 3, showcasing loss and accuracy metrics throughout the training period. Initial results with an unfiltered dataset yielded high accuracy and low loss rates on a test set that constituted 20% of the amino acid data, as shown in Figure 3a,b. Significant enhancements in model performance were recorded upon employing a filtered dataset—comprising the top 33 spectra for each amino acid—resulting in smoother learning curves and reduced loss, as shown in Figure 3c,d, indicative of a more efficient training regime. The model was then tested on an independent validation dataset obtained through additional experiments, and the prediction accuracies are shown in Figure 3e.

### 3.4. Evaluating the Predictive Accuracy of Machine Learning Models for Amino Acid Composition in Mixed Samples

**Figure 4:**
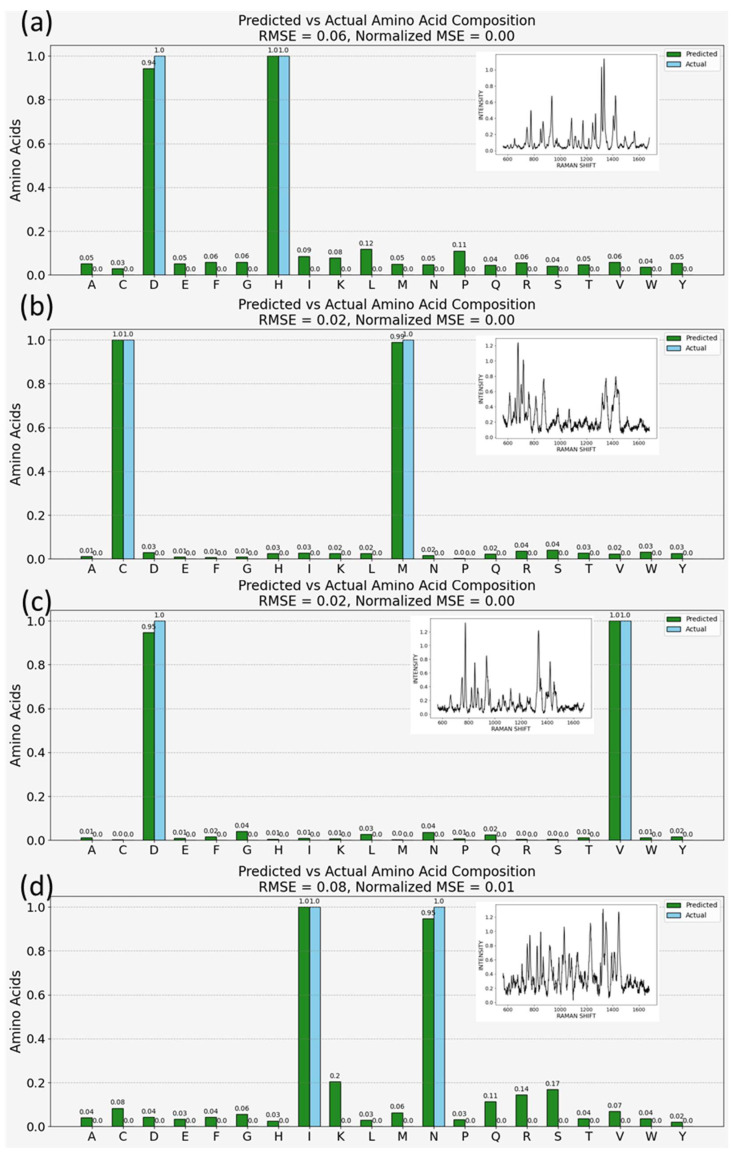
Model predictions for amino acid content in simulated peptide spectra (inset graphs) for the following doublets—(**a**) aspartic acid and histidine, (**b**) cysteine and methionine, (**c**) aspartic acid and valine, (**d**) isoleucine and asparagine [50,51,52].

Figure 4 showcases the model’s capacity to deduce amino acid compositions from simulated SERS spectra involving pairs of amino acids, such as aspartic acid and histidine, cysteine and methionine, aspartic acid and valine, and isoleucine and asparagine. The simulations were conducted on clean and pre-processed spectra, and the model accurately determined the constituents of these complex mixtures. Predictive outcomes, illustrated through green bars in Figure 4, matched well with the known compositions of these simulated peptides, marked by blue bars. In quantifying the model’s precision, root mean square error (RMSE) and mean squared error (MSE) were employed as the primary evaluative metrics. The RMSE values, under 0.1 or 10%, suggest a high degree of accuracy in the model’s predictions, resonating with the rigorous standards set for medical device evaluations.

### 3.5. Analysis of Real-Life, Disease-Relevant Proteins and Composition Predictions

**Figure 5:**
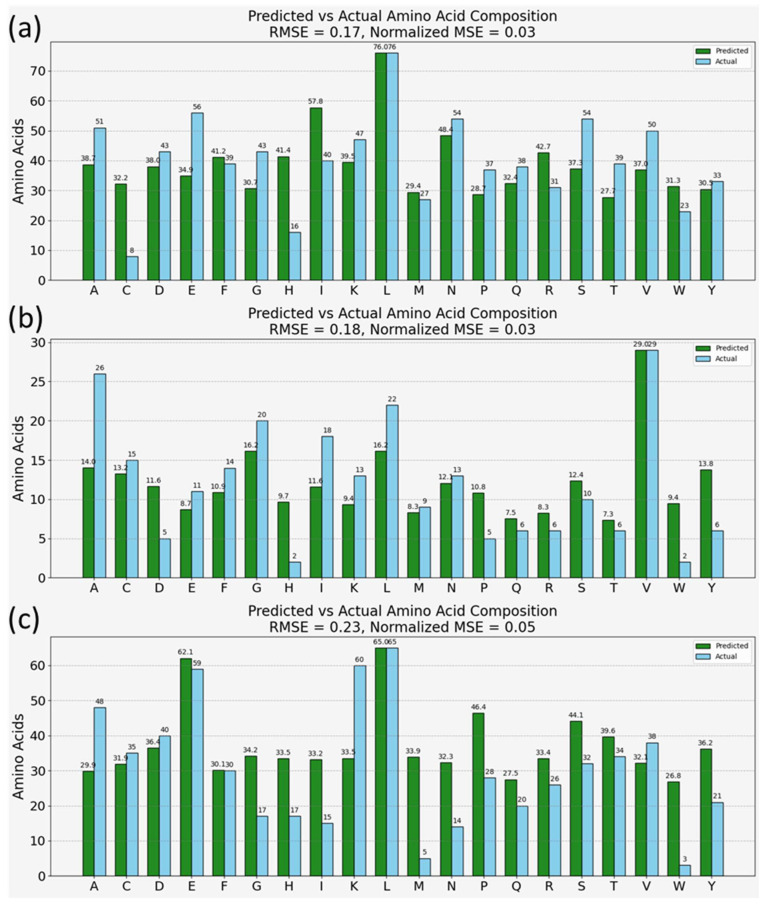
Model predictions on SERS spectra of commercially obtained proteins—(**a**) ACE2, (**b**) CD63 antigen, (**c**) BSA.

Figure 5 presents the model’s predictions for the amino acid compositions of three well-characterized proteins: bovine serum albumin (BSA), ACE2 protein, and CD63 antigen. These proteins were selected for their relevance to laboratory research, SARS-CoV-2 virus studies, and immune response investigations, respectively. The model’s predictions, depicted as green bars in Figure 5, were compared to known amino acid compositions from the Uniprot database, shown as blue bars. The comparison demonstrated a notably low normalized root mean square error (RMSE) across all three proteins, indicating a high level of accuracy in the model’s predictions. The model was also successful in identifying the most abundant amino acid within each protein sample.

## 4. Discussion

### 4.1. Analysis of SERS Spectra

In exploring the surface-enhanced Raman spectroscopy (SERS) spectra of the 20 amino acids, our initial findings present an intriguing juxtaposition between the averaged spectra derived from our laboratory experiments and those reported in existing literature. As depicted in Figure 1, the spectra we obtained (illustrated in blue) display some discrepancies when compared with spectra derived from previous studies [37,38] (shown in red). This variance underscores a pivotal aspect of SERS-based analyses: the outcome is heavily influenced by the experimental setup, including the spectrometer used, its operational parameters, and the substrate employed. These findings highlight the necessity of establishing our own spectral database to ensure accuracy and reliability in our analyses, acknowledging that spectra variability can arise from subtle differences in experimental conditions [48].

The initial set of results presents the averaged surface-enhanced Raman spectroscopy (SERS) spectra for each of the 20 amino acids. Figure 1 displays these spectra, with each representing an average of 75 individual spectra per amino acid. This comprehensive collection provides a foundational understanding of the characteristic spectral signatures of each amino acid, setting the stage for further analysis and model training. Notably, tryptophan is distinguished by its strong Raman bands around 1552 cm^−1^, which is attributed to the indole ring breathing mode, and a band near 760 cm^−1^, related to the C-H bending out of the plane of the indole ring [49]. Alanine, on the other hand, shows a characteristic peak around 1445 cm^−1^, associated with the CH_3_ deformation. Phenylalanine is characterized by Raman bands at approximately 1000 cm^−1^ and 1033 cm^−1^, corresponding to the breathing modes of the benzene ring [38,49]. These unique peaks are crucial for the identification and differentiation of these amino acids in SERS analysis. 

The CV values were calculated for the amino acid dataset, and they reveal a variable degree of reproducibility across the different amino acids. As seen in Figure 1, while several amino acids demonstrate CV values below the 10% benchmark, indicating high reproducibility, others exceed this threshold, suggesting areas for improvement in experimental consistency or specificity of peak selection. Though all CV values are below 20%.

In summary, by calculating the CV for the peak intensities of the ten major Raman bands across the measurements for each amino acid and comparing these values against a benchmark CV of less than 10%, we can quantitatively assess and argue the reproducibility of our experimental setup. This approach provides a clear, standardized metric for evaluating the consistency of our spectral data, ensuring the reliability of our findings. The nuanced discrepancies observed between our spectra and those from the literature reinforce the importance of a standardized experimental setup for reproducibility and reliability in SERS analyses. The utility of our findings lies in their reproducibility within a fixed experimental setup, as evidenced in Figure 1. The blue spectra, each an average of 50 measurements, underscore the rigor of our experimental process, highlighted by the clarity and consistency of the distinct peaks, with none being canceled out or averaged away. This level of reproducibility, quantitatively supported by the coefficient of variance values presented in the figure indicating minimal fluctuation, showcases the meticulousness of our methodology. For a more detailed visual comparison and to further affirm the reproducibility and accuracy of our data, supplementary data Sections S2 and S4 feature a side-by-side spectral comparison for all amino acids, emphasizing that within our experimental conditions, the spectral data remains remarkably consistent.

### 4.2. Unsupervised Clustering of Amino Acid Spectra

Subsequent results focused on clustering analysis using t-SNE. In Figure 2a, we showcased the formation of 20 distinct clusters through unsupervised clustering of 2420 spectra, with each amino acid contributing 121 spectra obtained via Raman mapping. This visualization illustrated the natural groupings within the spectral data. In Figure 2b, we presented enhanced clustering results achieved by sorting the data based on the signal-to-noise ratio and selecting the 33 best spectra for each amino acid. This refinement in data selection further improved the clarity and distinction of the clusters. Each cluster correlates with one of the 20 amino acids in the study, suggesting a high degree of specificity in the SERS spectral data for amino acid identification. This unsupervised clustering not only reinforces the reliability of SERS as an analytical tool for amino acid differentiation but also validates the potential of using unsupervised machine learning techniques in processing complex spectral data.

A critical aspect of our methodology was the data cleaning and preprocessing phase. Initial clustering of 121 spectra per amino acid produced discernible clusters; however, we observed that the inclusion of all spectra introduced a degree of variability likely attributable to a lower signal-to-noise ratio in some measurements. By implementing a data cleaning process that filtered out these spectra, we refined our dataset to the 33 highest-quality spectra per amino acid. This step was crucial in reducing noise and improving the clarity of our data, thereby enhancing the subsequent training and performance of our neural network models.

The impact of this preprocessing was significant; the neural network models trained on this cleaner, high signal-to-noise ratio dataset demonstrated superior performance in predicting amino acid composition within protein samples. This underscores the importance of rigorous data preprocessing in spectroscopic studies, where the quality of the input data is paramount. By focusing on high-quality, high-signal-to-noise spectra, we were able to train models that could more accurately interpret the complex SERS spectra of proteins and estimate their amino acid compositions.

This distinction between internal (within each amino acid data) and external (between different amino acids’ data) variability is crucial. It demonstrates beyond reasonable doubt that, within the confines of our experimental setup, amino acids produce highly distinguishable SERS spectra. The external variability between different amino acids’ spectra far exceeds the minor internal variations observed within repetitions of the same amino acid. This finding is instrumental in validating the use of SERS combined with machine learning for accurate amino acid and protein analysis. It highlights the importance of considering both the quality of spectral data and the analytical techniques employed to interpret such data.

Hence, our study not only showcases the importance of data preprocessing in enhancing the clarity and reliability of clustering analysis but also confirms the distinctiveness of amino acid spectra despite internal fluctuations. By demonstrating that intra-amino acid spectral variations are minimal compared to the variations between different amino acids, we affirm the robustness of our approach. This work sets the stage for further advancements in proteomics, offering a scalable and repeatable method for amino acid and protein characterization using SERS spectra.

### 4.3. Model Training and Performance on Test Data

Figure 2b establishes the distinguishability between amino acid data, but it does not allow us to achieve what we have set out to do—train the machine learning models on this data to estimate the composition of amino acids in complex molecules, such as proteins. In the t-SNE clustering model used above, due to the coordinate transformation, we are making the physical significance of the model output difficult to decide. Hence, t-SNE is utilized only to demonstrate clear distinctions between the complex amino acid datasets, which is a necessary precursor to the goal of this paper. Subsequently, we hypothesize that a classification model would be more suitable for depicting amino acid compositions of proteins from their SERS spectra. Specifically, when the model is well-trained on a diverse dataset of amino acid spectra, these confidence levels can be interpreted as reflective of the relative abundance of each amino acid in the sample, assuming that higher spectral contributions from an amino acid result in higher probabilities.

The neural network model’s training process is illustrated in Figure 3, which shows the loss and accuracy curves over the training duration. For the unfiltered dataset, the accuracy and loss curves (Figure 3a,b) indicated that the model performed commendably, as demonstrated by the high accuracy and low loss on the test dataset, which comprised 20% of the amino acid data.

However, a notable improvement was observed when the model was trained with the filtered dataset, where the 33 highest-quality spectra per amino acid were used. The corresponding accuracy and loss curves (Figure 3c,d) revealed smoother convergence and a lower loss, indicating a more stable and effective training process. This improvement can be attributed to the refinement of input data through rigorous preprocessing, which filtered out spectra with lower signal-to-noise ratios, thereby enhancing the quality of the dataset.

The model’s enhanced performance with the filtered dataset, as depicted in Figure 3c,d, showcases the value of high-quality input data in neural network training. Noise reduction in the preprocessing stage ensures that the spectral features are distinct and characteristic, allowing the model’s attention mechanism to accurately focus on informative aspects of the spectra. This leads to a reduction in overfitting risks, as the model is less likely to interpret noise as a part of the signal, thereby enhancing its ability to generalize to new data. Moreover, the stability of the training process is improved, reflected in the smoother convergence of the loss and accuracy curves, which facilitates optimization and may result in computational efficiency gains. The preprocessing of data, by filtering out low-quality spectra, not only contributes to a more robust learning process but also underscores the critical role of data quality in the interpretation of complex SERS spectra for proteomic analysis.

The attention mechanism’s ability to discern and prioritize critical features in spectral data was augmented by the high quality of the input data. This synergy between sophisticated model architecture and meticulous data preprocessing underscores the importance of data quality in the field of machine learning, especially in applications where precision is paramount, such as in the interpretation of complex SERS spectra for proteomic analysis.

This section demonstrates the crucial role of data quality and preprocessing in training neural network models for the precise interpretation of complex SERS spectra, underscoring the paper’s theme that advanced machine learning techniques, when applied to high-quality, carefully pre-processed datasets, can accurately estimate amino acid compositions in proteins, reflecting the broader potential of these methods in proteomic analysis.

Furthermore, the model performance was validated on an independent dataset of amino acids obtained through separate experiments. The dataset contained 50 spectra for each amino acid, and predictions were made for each spectrum. The average values of prediction accuracies are presented in Figure 3e, and most accuracies are above 95%, with the average across the entire validation dataset being 95.4%. Hence, the performance on a completely external, unique, and diverse dataset shows that the model trained on all 20 amino acids can identify each of the amino acids independently. This strong validation performance shows that the model has effectively been trained on the amino acid data and can be used for the next step in our study—predicting amino acid composition in proteins.

### 4.4. Evaluating the Predictive Accuracy of Machine Learning Models for Amino Acid Composition in Mixed Samples

Figure 4 presents the model’s capability to predict amino acid compositions from simulated spectra. This simulation involved combining SERS spectra of amino acid pairs: aspartic acid and histidine (Figure 4a), cysteine and methionine (Figure 4b), aspartic acid and valine (Figure 4c), and isoleucine and asparagine (Figure 4d). The inset figures present examples of the simulated spectra on which the model makes amino acid predictions. The model, trained on clean and pre-processed individual amino acid spectra, accurately identifies the constituents within these complex mixtures. As mentioned above, this is a 20-class classification task, and the predictions are probabilistic. In the figure, the green bars correspond to the model output, and the blue bars are the actual composition of the simulated peptide. The height of the green bars represents the model’s confidence. In each case, the model correctly identifies the constituent amino acids, with the highest peaks aligning with the amino acids known to be present in the doublets. The ability to predict amino acid compositions from SERS spectra of mixtures indicates that this tool can be utilized for inferring the presence and concentration of amino acids in protein samples, which is shown below.

In our analysis, the root mean square error (RMSE) and mean squared error (MSE) were selected as the primary metrics for evaluating the precision of our model due to their ability to effectively capture the average magnitude of errors between predicted and actual values. RMSE, by taking the square root of the average of squared differences, provides a directly interpretable measure in the same units as the original measurements, making it exceptionally useful for assessing the model’s accuracy in predicting amino acid compositions. Meanwhile, MSE offers a squared quantification of the average error, emphasizing larger errors more significantly than smaller ones, which is crucial for fine-tuning our model’s performance by identifying and minimizing large prediction errors. Quantitatively, the model’s precision, with a root mean square error (RMSE) of less than 0.1 or 10%, demonstrates high quantitative accuracy, akin to the stringent standards observed in medical device evaluations, such as continuous glucose monitoring (CGM) systems in diabetes management. In this context, the mean absolute relative difference (MARD), a common accuracy measure where values below 10% are considered excellent [50], is comparable to our model’s performance. Regulatory standards from the U.S. Food and Drug Administration (FDA) and the International Organization for Standardization (ISO) provide further benchmarks: ISO 15197:2013 (updated in 2021) requires 95% of glucose measurement results to be within ±15 mg/dL for concentrations <100 mg/dL and within ±15% for concentrations ≥100 mg/dL [51], while the FDA mandates similar clinical evaluations, accuracy comparisons, and user testing for CGM devices and blood glucose meters [52]. Our model’s achievement highlights its potential reliability and accuracy for scientific and medical applications, aligning with the precision criteria of these regulatory evaluations.

Applying a similar emphasis on accuracy to our model, achieving an RMSE of less than 0.1 in predicting amino acid compositions from complex SERS spectra signifies not only the model’s computational efficiency but also its potential applicability in precise biochemical analyses. This level of accuracy, comparable to the stringent criteria used in evaluating medical devices, underscores the reliability of our approach for detailed proteomic studies and other applications requiring fine-grained molecular analysis. Hence, based on the results in this section, at least for the limited number of variations we produced, the ML model successfully finds agreement with the true composition of the samples.

### 4.5. Analysis of Real-Life, Disease-Relevant Proteins and Composition Predictions

Figure 5 illustrates the model’s prediction results for three well-characterized proteins: Bovine Serum Albumin (BSA), a commonly used protein in various lab applications due to its stability and lack of interference within biological reactions; ACE2 protein, known for its role as the receptor for the SARS-CoV-2 virus entry into cells; and CD63 antigen, typically associated with cellular processes like signal transduction and development of immune responses. The model’s predictions for these proteins are represented by green bars and contrasted with the actual amino acid compositions extracted from the Uniprot [53] database (shown in blue bars) within the double bar plots of Figure 5a–c.

The normalized root mean square error (RMSE) is notably low in each case, suggesting a high level of accuracy in the model’s predictions. Furthermore, the model correctly identifies the most abundant amino acid within each protein, underscoring its efficacy. However, there are deviations observed between the predicted and actual compositions. From a machine learning perspective, these deviations could stem from the absence of peptide bonds and the complexities of protein conformations in the training dataset, which are inherently present in the protein samples. Biochemically, variations in peak intensities due to protein conformations, which alter the physical and chemical environment of amino acid residues, are not represented in the training set of free amino acids [1,4]. Such factors highlight the intricacies of protein structures that may affect SERS spectra, including the orientation of amino acids, their interactions within the protein matrix, and modifications that occur post-translationally, all of which can contribute to the observed discrepancies. These points suggest avenues for further refining the training datasets and the model to better account for the complexities of protein structures in SERS-based proteomic analysis. Nevertheless, the overall results are encouraging, demonstrating the model’s substantial potential as a tool for proteomic analysis. It suggests that, with further refinement to incorporate the diverse aspects of protein biochemistry and structure, such models can be instrumental in advancing non-invasive, rapid proteomic techniques. 

Additionally, Raman spectroscopy is widely recognized for its ability to provide detailed chemical information, particularly about the atomic and molecular bonds within a substance. However, the ability of the model to closely approximate the amino acid composition of complex proteins from their SERS spectra highlights how this chemical information, typically analyzed at the atomic or molecular level, is preserved, and interpreted at the macromolecular scale in SERS spectra. Proteins, which are complex assemblies of amino acids, themselves compounds of intricately arranged atoms, present a higher order of structural organization, beyond atomic bonding. The SERS technique (due to its chemical characterization ability) not only delineates the various bonds and atomic interactions within amino acids but also retains holistic information on how many of these amino acids are present within proteins. Thus, this study demonstrates that SERS is not limited to identifying individual chemical bonds; it effectively maps out the higher-order structures of amino acids as they are configured in proteins. This insight underscores the potential of SERS, revealing not just the atomic or molecular specifics but also how groups of these entities assemble to form larger, functionally significant biological structures. 

### 4.6. Interpretation and Implications of Findings

The utilization of SERS spectra of 20 amino acids has enabled us to explore the predictive capabilities of the transformer neural networks in identifying the predominant proteins within the sampling volume of SERS. The distinctiveness in the spectral data, as demonstrated through our comparative analysis between laboratory-derived spectra and those from existing literature (Figure 1), underscores the influence of experimental setups on the outcomes of SERS-based analyses. This variance necessitates the establishment of a specific spectral database to ensure the reliability and accuracy of analyses, addressing a crucial aspect of SERS-based studies.

The quantitative reproducibility assessment of our spectral data, employing the coefficient of variation (CV) across the characteristic Raman peaks, underlines the imperative for precision in experimental consistency. Although some amino acids demonstrated high reproducibility within the benchmark of less than 10% CV, indicating the robustness of our experimental setup, variations suggest areas for refinement in experimental conditions or peak selection criteria. These findings not only affirm the reproducibility within our standardized setup but also highlight the nuanced discrepancies that could emerge from subtle differences in experimental protocols.

The clustering analysis (in Figure 2), particularly the enhanced distinction achieved through data preprocessing, further illustrates the specificity and reliability of SERS as an analytical tool for amino acid differentiation. By demonstrating the significant improvement in model performance with cleaner, high-quality datasets, our study emphasizes the importance of data quality in the interpretation of complex SERS spectra. This not only validates the potential of machine learning in processing spectral data but also reinforces the need for rigorous data preprocessing in spectroscopic studies.

Moreover, our findings from the predictive accuracy assessment of amino acid compositions in mixed samples (Figure 4), alongside the analysis of real-life, disease-relevant proteins (Figure 5), offer compelling evidence of the model’s utility in proteomic analysis. The model’s ability to closely approximate the amino acid composition of proteins from their SERS spectra, despite observed deviations, underscores its potential in non-invasive, rapid proteomic techniques. These deviations, potentially stemming from the absence of peptide bonds or protein conformation complexities in the training dataset, highlight areas for future refinement.

The methodological advantages of combining SERS with AI technologies extend beyond the analytical improvements; they encompass the potential for non-invasive or minimally invasive sample analysis, rapid results delivery, and high specificity and sensitivity, which could revolutionize molecular diagnostics and analysis in biological laboratories. However, the interpretability of deep learning models and the uniqueness of SERS spectral features pose notable challenges, emphasizing the need for continued research to enhance the precision and applicability of these methods.

Furthermore, while our study showcases the potential of SERS for detailed molecular analysis, it’s important to recognize the complementary role it plays alongside established proteomic techniques such as mass spectrometry. Sophisticated mass spectrometry methods, including tandem MS and high-resolution MS, remain indispensable for the detection, identification, and quantification of proteins, peptides, and amino acids due to their depth of analysis and precision. Our findings suggest that SERS, augmented by AI-driven spectral decomposition, offers a non-invasive, rapid approach that could enhance the proteomics toolkit, especially in scenarios where traditional MS may be limited by sample preparation requirements or the need for direct functional state information of proteins. 

### 4.7. Discussion on Methodological Advantages and Limitations 

From the clinical perspective, should this technique become successful, it harbors a series of significant advantages that could benefit the landscape of molecular diagnostics and analysis in biological laboratories. Firstly, the SERS + AI method stands out for its non-invasiveness or minimal invasiveness, offering a stark contrast to the conventional invasive sample collection methods. Secondly, it promises rapid analysis and results delivery, crucial in clinical settings where timely decision-making can be crucial. Thirdly, the technique’s potential for high specificity and sensitivity could lead to more accurate diagnoses. Fourthly, its adaptability to various sample types broadens its application range, making it a versatile tool in the clinical arsenal. Finally, a major benefit of an ML-based approach is the digitalization of spectral data, which enables the creation of a permanent record of molecular fingerprints. This not only streamlines the analytical process by reducing the need for repeated reagent use in each test—a common requirement in traditional methods—but also ensures the perpetual availability of molecular fingerprints for scalable and sustainable research.

A key advantage of this technique over traditional methods used in biological labs lies in its information richness. Whereas typical biological assays, like antigen–antibody tests, might rely on a single dimension of data, our approach taps into a wealth of over a thousand dimensions obtained in the spectral data. This richness in data dimensions offers a fundamentally more nuanced view of the molecular composition of samples. However, the advantage of information richness comes with its own set of challenges. While having a multitude of data dimensions allows for a detailed analysis, it also raises the question of whether this information primarily reflects the target molecules or if it’s influenced by other factors. The crux of leveraging such information-rich technologies lies in their ability to discern and accurately identify the molecules of interest, amidst a backdrop of potentially confounding variables, such as the presence of impurities. Extracting the relevant factors with a high correlation to the target condition or molecule, amidst a sea of rich but complex data, can indeed be challenging. Another challenge is the interpretability of deep learning models, often described as a “black box” due to their opaque nature [54,55]. In scientific contexts, it’s crucial to understand the rationale behind model predictions, but explaining and interpreting these predictions can be complex. Additionally, while the choice of neural network architecture can influence accuracy, the physics of SERS signal intensity at the molecular level may result in non-unique spectral features [2,24,56]. These challenges require careful consideration and ongoing research.

The application of our findings may contribute positively to several areas, particularly in enhancing the efficiency of proteomic analysis. The combination of SERS and AI techniques suggests a pathway toward more precise, non-invasive diagnostics, potentially enabling earlier detection and ongoing monitoring of diseases at a molecular level. The specificity and sensitivity demonstrated by our model also indicate its usefulness in identifying biomarkers, which could inform drug development and tailored treatment plans. Moreover, the technique’s adaptability to different sample types, coupled with the reduction in preparation needs, could simplify laboratory workflows and reduce operational costs, reflecting a logical step forward in both clinical and research environments.

Finally, our study reinforces the notion that SERS spectra are rich in biochemical content information. This finding is crucial as it suggests that SERS can be a reliable tool for detailed molecular analysis, capturing subtle biochemical changes that other techniques might miss.

## 5. Conclusions

Our study underscores the potential of combining Surface-Enhanced Raman Spectroscopy (SERS) with transformer neural networks as a supplementary tool for proteomic analysis, particularly in predicting protein compositions from SERS spectra. This novel approach could complement established techniques such as mass spectrometry, offering advantages in scenarios where traditional methods are limited by sample preparation requirements or the need for direct functional state information. We highlight the importance of refining our model for improved accuracy and interpretability and emphasize the need for ongoing efforts to enhance SERS and AI methodologies in molecular diagnostics. Nevertheless, we acknowledge the challenges ahead, including the critical need for model interpretability and the adaptation to the dynamic nature of molecular structures. This study further illustrates that the information encapsulated in SERS spectra encompasses key biochemical information from complex bioparticles. The ability of our model to predict amino acid compositions from these spectra indicates its capacity to reveal intricate biochemical insights that could be crucial for understanding the molecular basis of diseases and facilitating the development of targeted therapies. Looking forward, we advocate for collaborative research to further explore the capabilities of SERS in molecular analysis, focusing on expanding the spectral database for proteins and peptides, refining data preprocessing techniques, and improving the model’s ability to handle complex biological mixtures.

## Data Availability

Data are contained within the article and the Supplementary File.

## Supplementary Materials

The following supporting information can be downloaded at: https://www.mdpi.com/article/10.3390/bioengineering11050482/s1, Section S1: Transformer Model Architecture; Section S2: Demonstrating Reproducibility of SERS Spectra for Each Amino Acid; Section S3: SEM Images of SERS Substrate, Section S4: Amino Acid Validation Data for the Trained Model.
