# Supplementary material for: AI-Driven Spectral Decomposition: Predicting the Most Probable Protein Compositions from Surface Enhanced Raman Spectroscopy Spectra of Amino Acids
Source: Bioengineering (Basel). 2024 May 12;11(5):482. doi: 10.3390/bioengineering11050482 (PMC11117800; doi:10.3390/bioengineering11050482)

AI-driven Spectral Decomposition: Predicting the Most Probable Protein Compositions from SERS Spectra of Amino Acids

Siddharth Srivastava, Nehmat Sandhu, Jun Liu, Ya-Hong Xie*

Supplementary Data

S1. Transformer Model Architecture

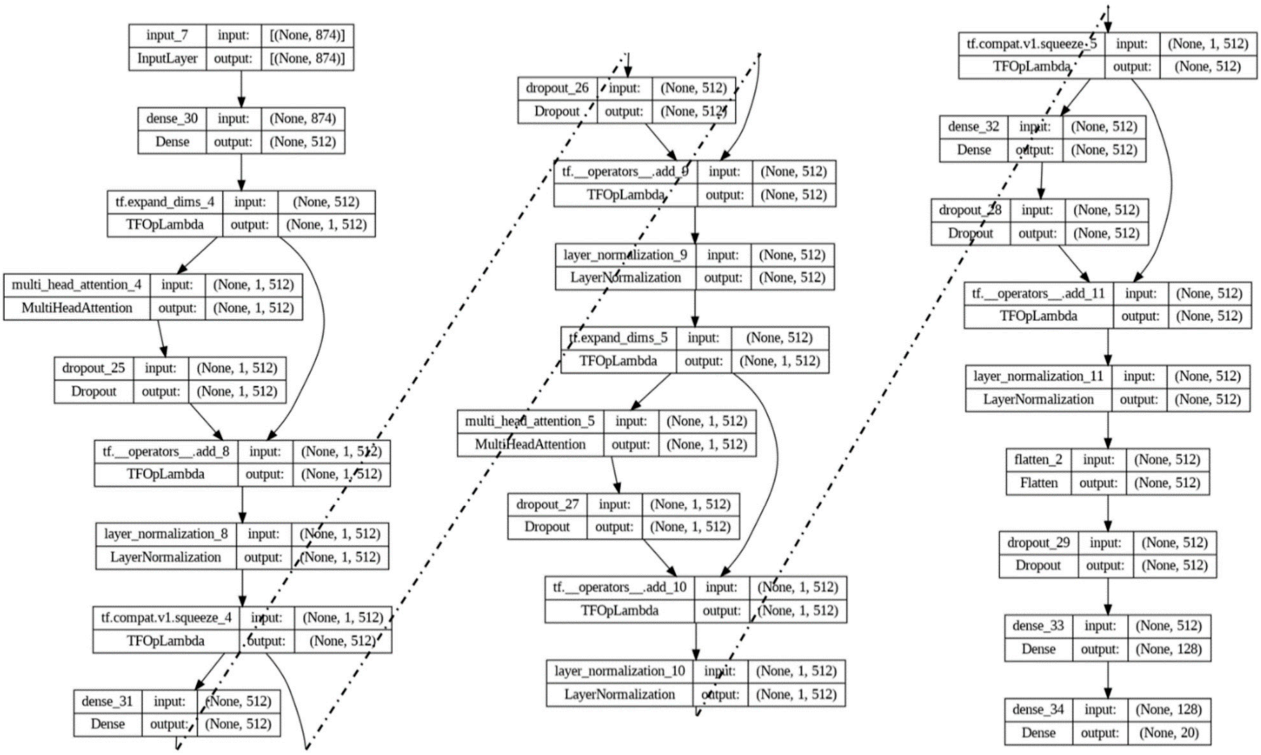

This neural network model incorporates a Transformer architecture to analyze and predict amino acid compositions from spectral data. The model's design leverages the power of attention mechanisms, enabling it to focus on the most relevant parts of the input data for making predictions.

Model Overview:

The model begins with an input layer designed to accept spectral data, which has been preprocessed and scaled. The input shape is determined by the number of features in the spectral data, ensuring flexibility to accommodate various datasets.

Following the input layer, the model employs a dense layer with 512 neurons, activated by the ReLU function. This dense layer serves to project the input features into a higher-dimensional space, where complex relationships in the data can be more easily learned.

Transformer Blocks:

At the core of the model are the Transformer blocks, which are applied to the output of the initial dense layer. Each Transformer block consists of two key components: a multi-head attention mechanism and a position-wise feedforward network, both of which include dropout for regularization and are wrapped with layer normalization to stabilize the learning process.

1. <u>Multi-Head Attention:</u> This mechanism allows the model to focus on different parts of the input sequence, considering multiple representations at different positions simultaneously. It

helps the model capture various aspects of the spectral data, enhancing its ability to understand complex patterns.

2. <u>Position-Wise Feedforward Network:</u> Each attention output is then passed through a feedforward neural network, applied identically to each position. This network further processes the data, allowing for the learning of more abstract representations.

The model utilizes multiple Transformer blocks in sequence, enhancing its capability to capture intricate dependencies in the data. The exact number of Transformer blocks can be adjusted based on the complexity of the task and the dataset.

Output Processing:

After passing through the Transformer blocks, the model's output is flattened, making it suitable for the final classification layers. A dropout layer follows the flattening step to reduce overfitting by randomly setting a portion of the input units to zero during training. The subsequent dense layer, activated by ReLU, serves as an additional processing step before the final output.

The output layer consists of 20 neurons, corresponding to the 20 standard amino acids, activated by the sigmoid function. This setup enables the model to predict the presence and relative abundance of each amino acid in the spectral data, outputting a probabilistic distribution across all amino acids.

Training Procedure:

The model is compiled with the Adam optimizer, a popular choice for deep learning models due to its adaptive learning rate capabilities. It uses binary crossentropy as the loss function, suitable for multi-label classification tasks where each class is predicted independently. The training process is monitored with early stopping and model checkpointing based on validation loss, ensuring that the best-performing model is saved without overfitting to the training data.

In summary, this Transformer-based neural network model represents a sophisticated approach to analyzing spectral data for amino acid composition prediction. Its design leverages the strengths of attention mechanisms and deep learning to capture complex patterns in the data, offering a powerful tool for proteomic analysis.

S2. Demonstrating Reproducibility of SERS Spectra for Each Amino Acid

1. Alanine | Coefficient of Variation (CV) = 11.13%

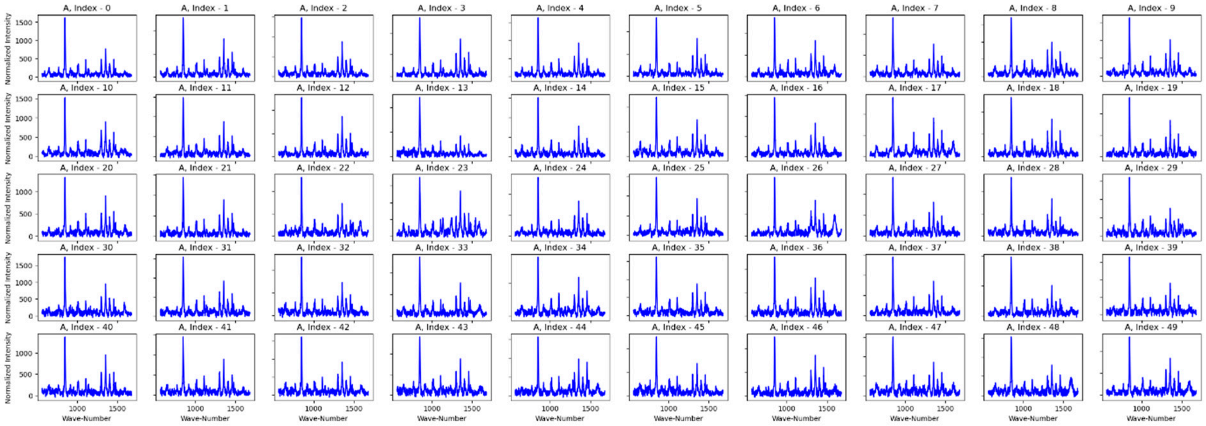

2. Cysteine | Coefficient of Variation (CV) = 19.03%

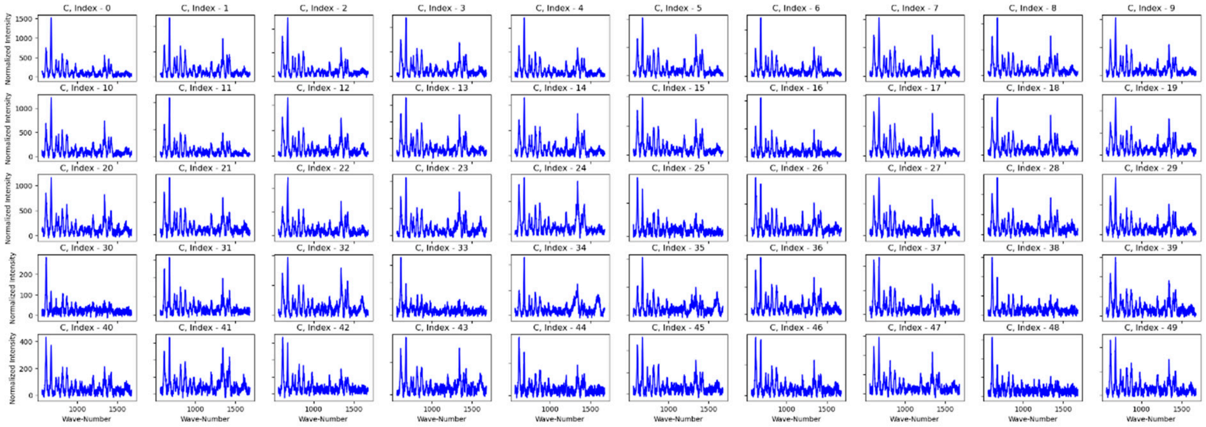

3. Aspartic Acid | Coefficient of Variation (CV) = 16.41%

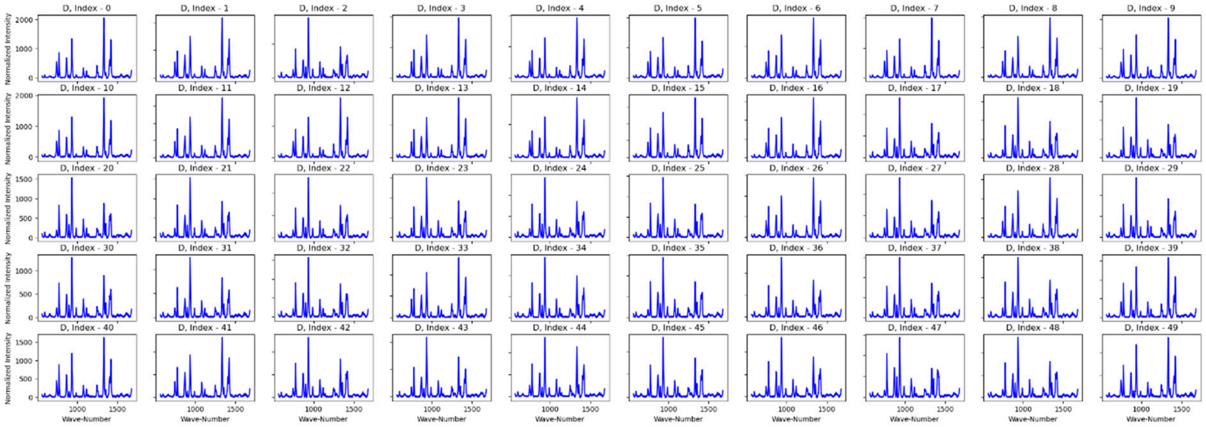

4. Glutamic Acid | Coefficient of Variation (CV) = 7.72%

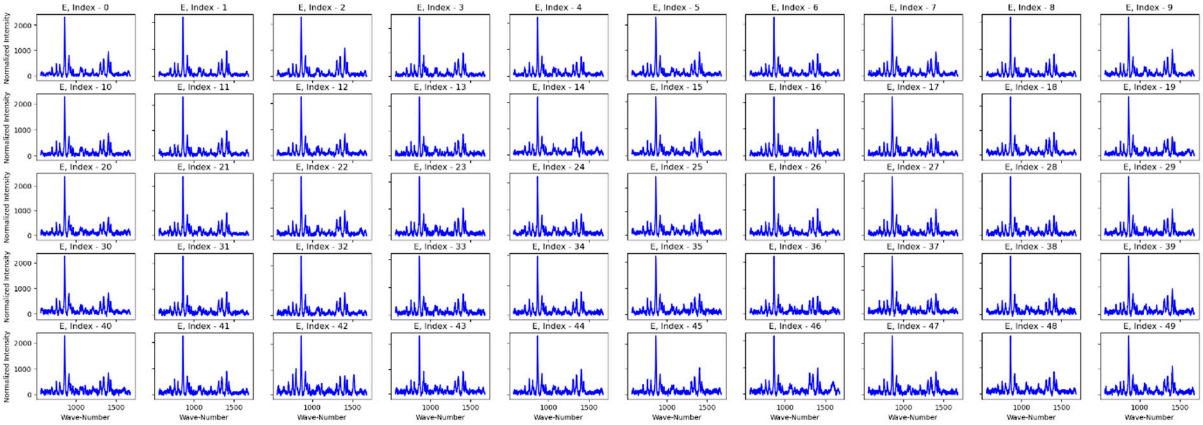

5. Phenylalanine | Coefficient of Variation (CV) = 7.53%

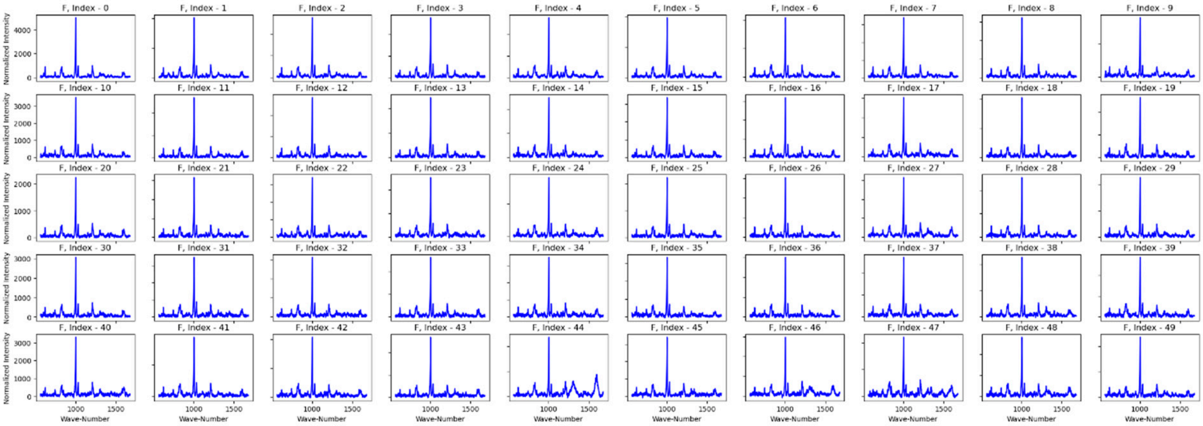

6. Glycine | Coefficient of Variation (CV) = 10.25%

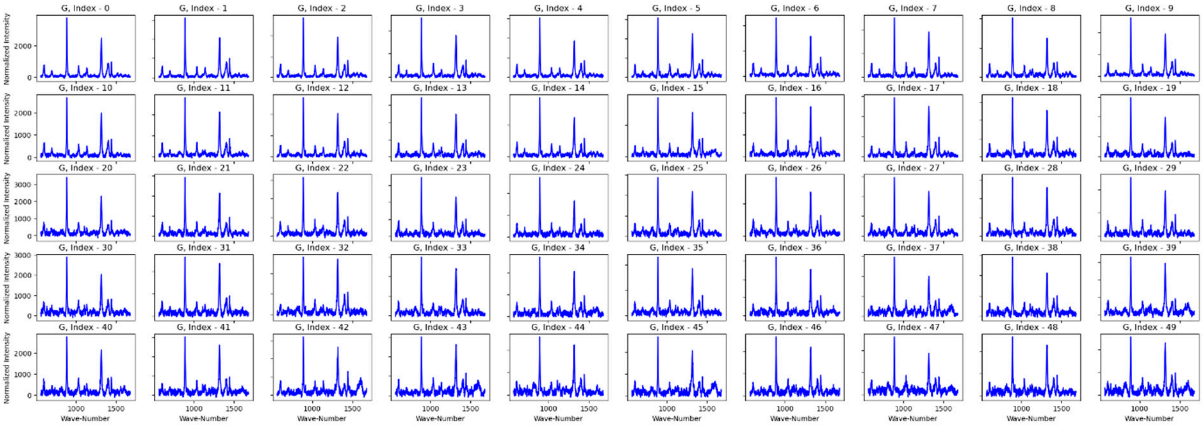

7. Histidine | Coefficient of Variation (CV) = 11.35%

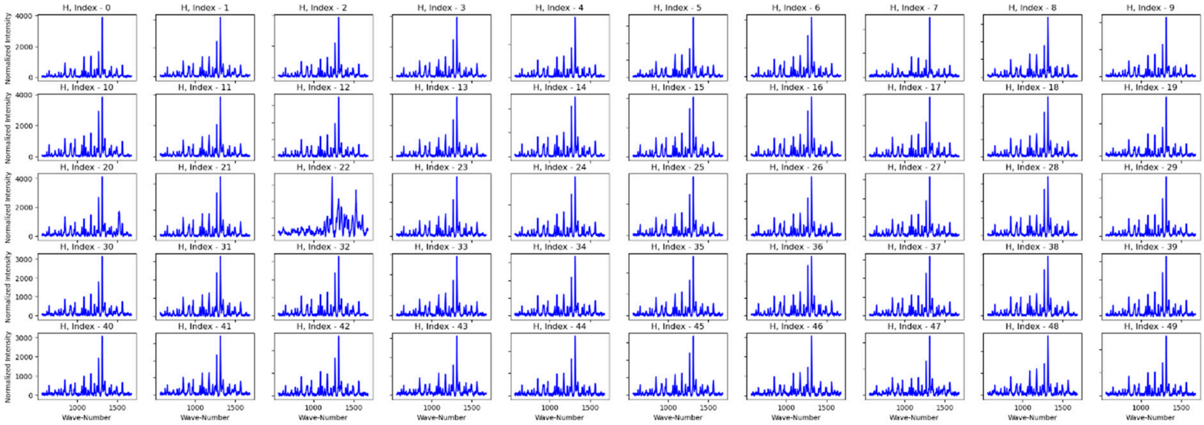

8. Isoleucine | Coefficient of Variation (CV) = 16.5%

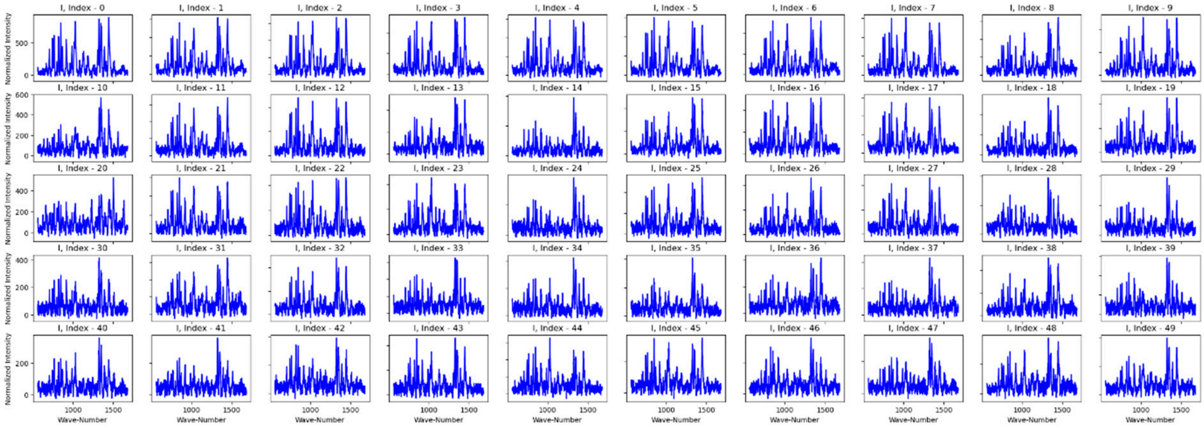

9. Lysine | Coefficient of Variation (CV) = 13.5%

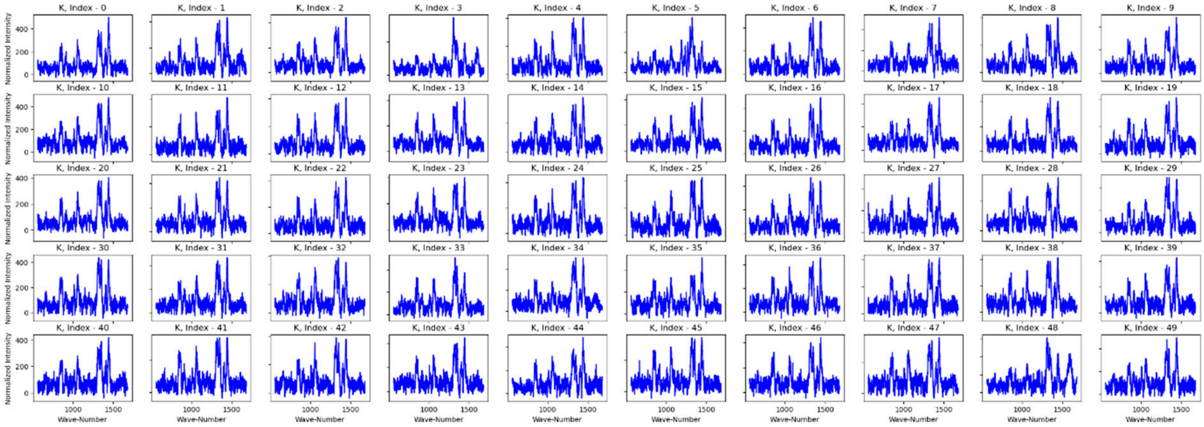

10. Leucine | Coefficient of Variation (CV) = 13.9%

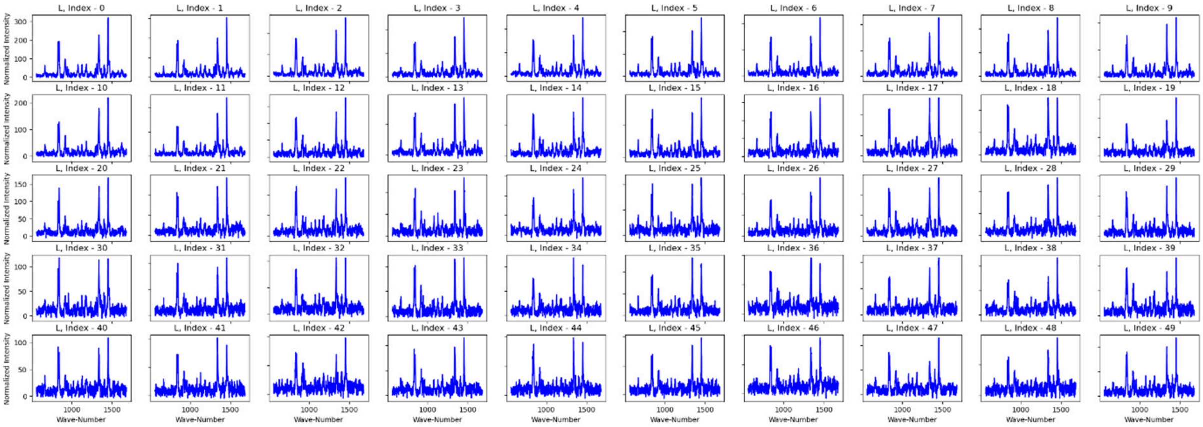

11. Methionine | Coefficient of Variation (CV) = 7.53%

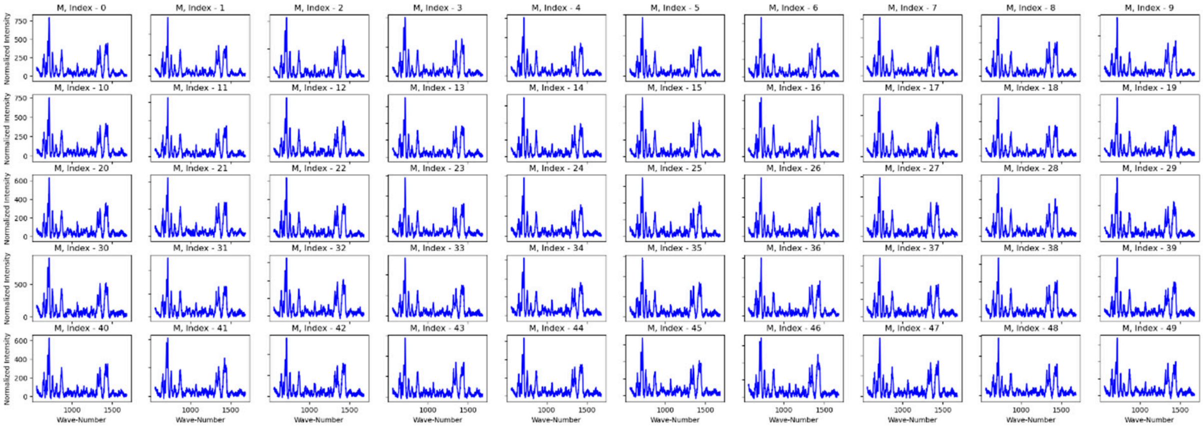

12. Asparigine | Coefficient of Variation (CV) = 13.27%

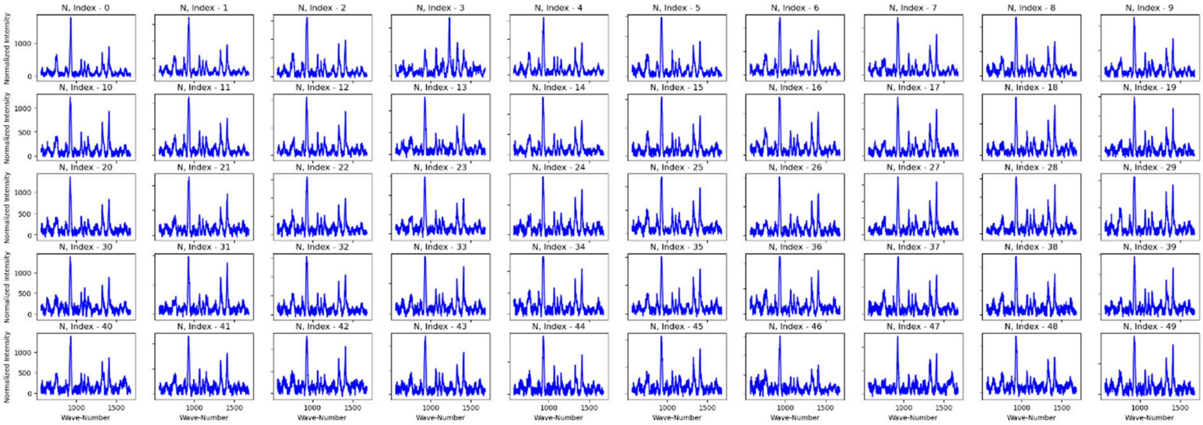

13. Proline | Coefficient of Variation (CV) = 9.75%

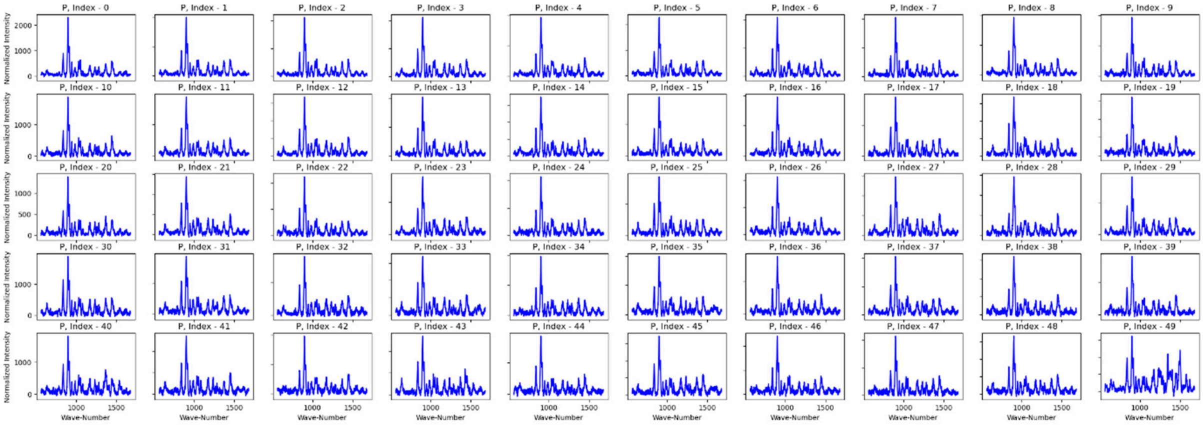

14. Glutamine | Coefficient of Variation (CV) = 3.59%

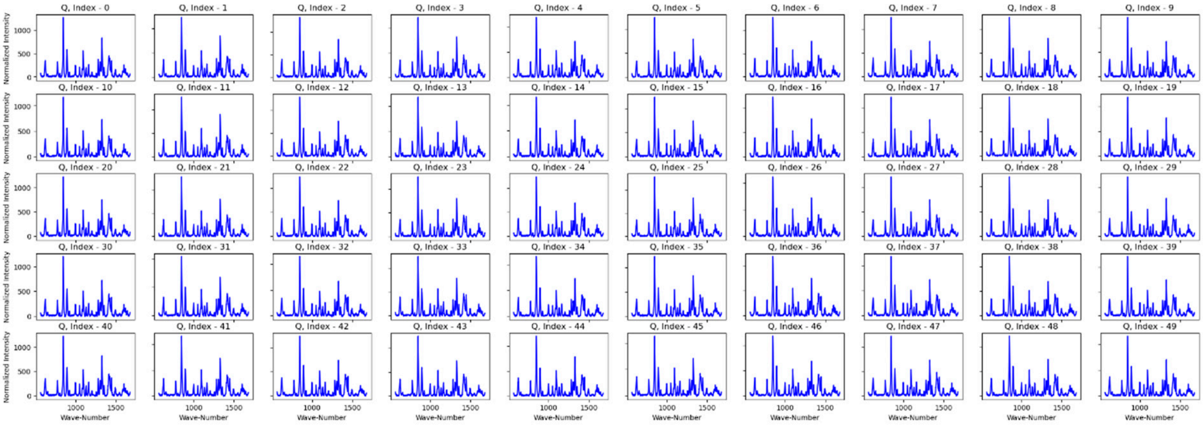

15. Arginine | Coefficient of Variation (CV) = 18.0%

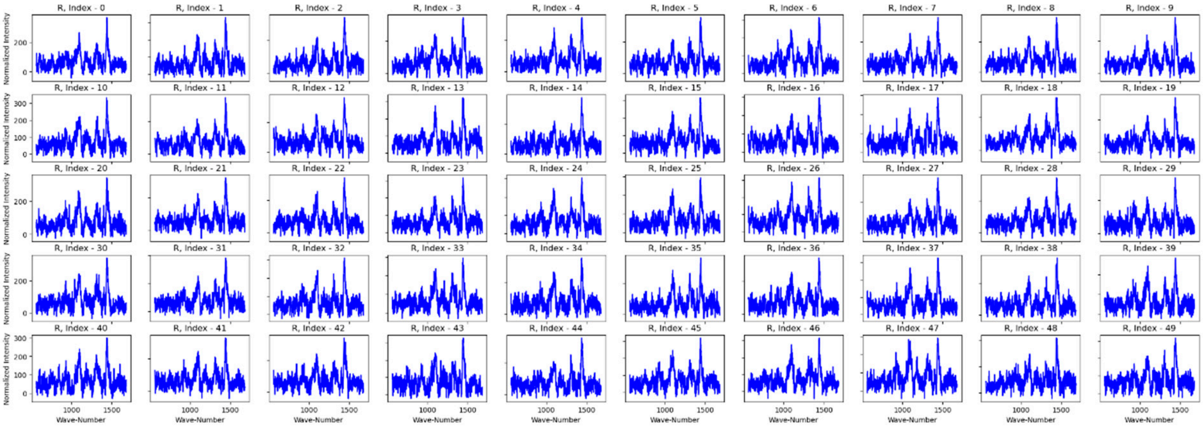

16. Serine | Coefficient of Variation (CV) = 6.42%

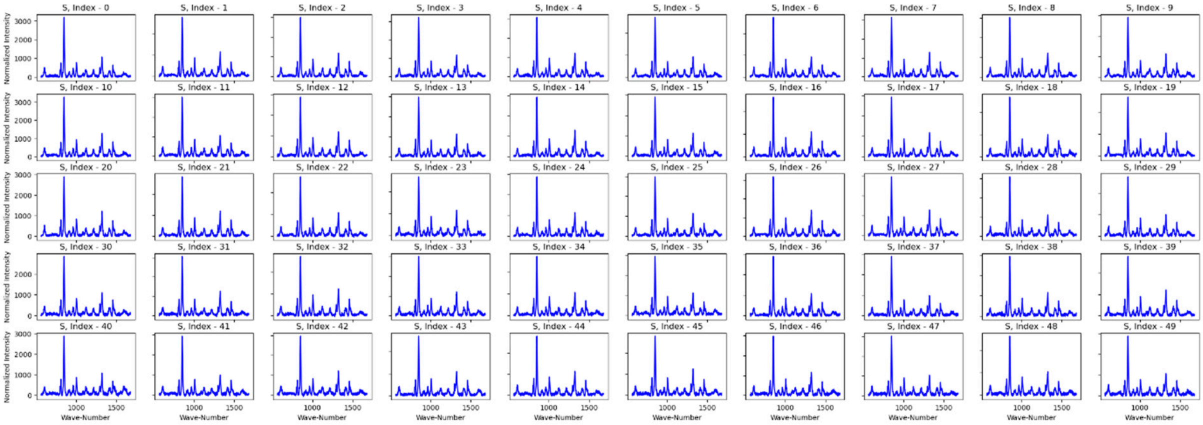

17. Threonine | Coefficient of Variation (CV) = 9.33%

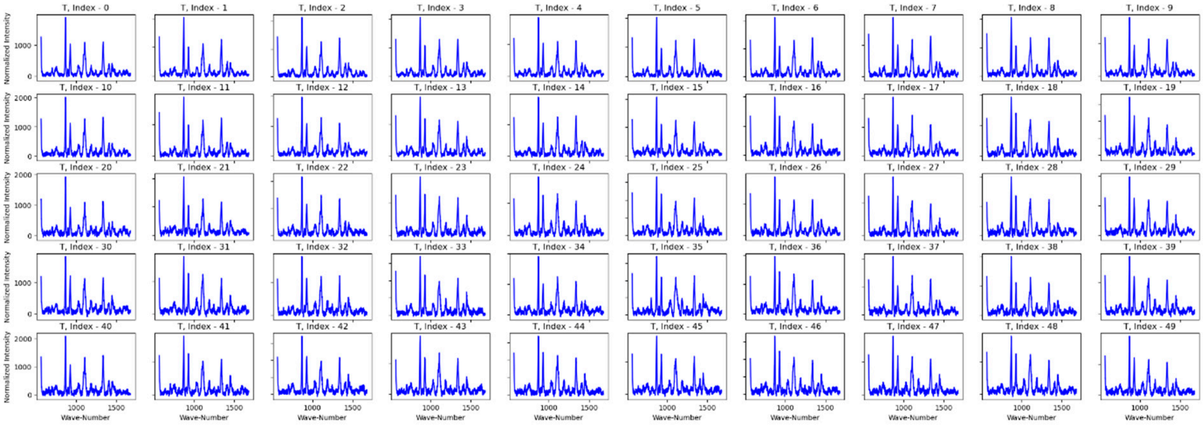

18. Valine | Coefficient of Variation (CV) = 8.87%

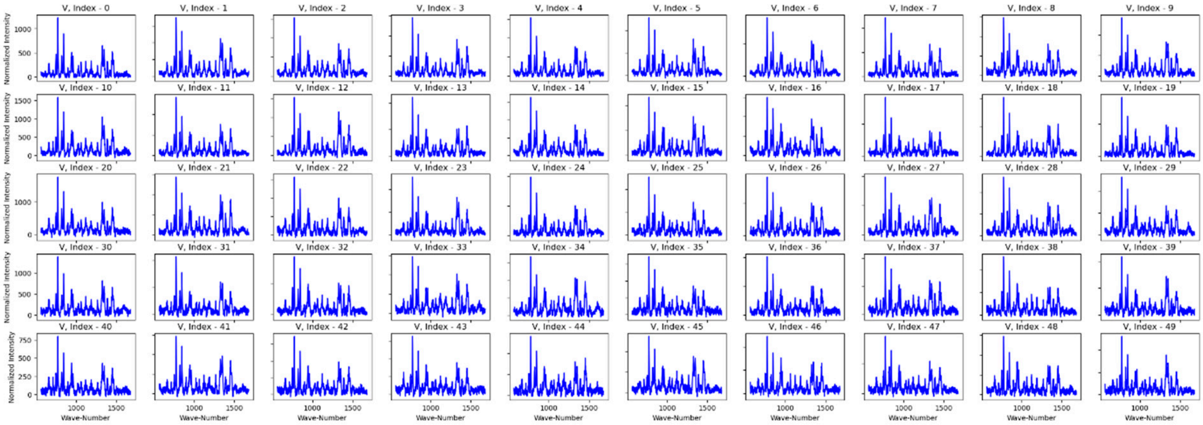

19. Tryptophan | Coefficient of Variation (CV) = 7.38%

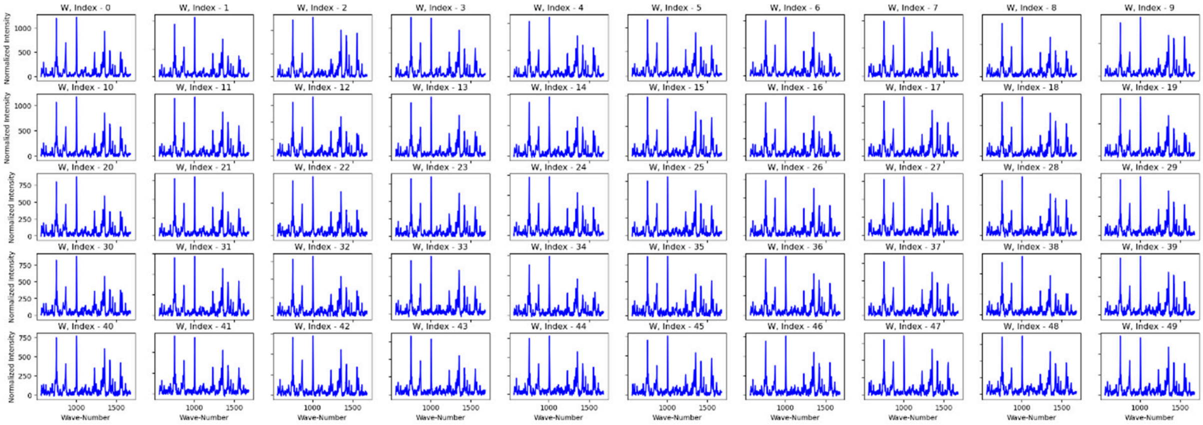

20. Tyrosine | Coefficient of Variation (CV) = 6.08%

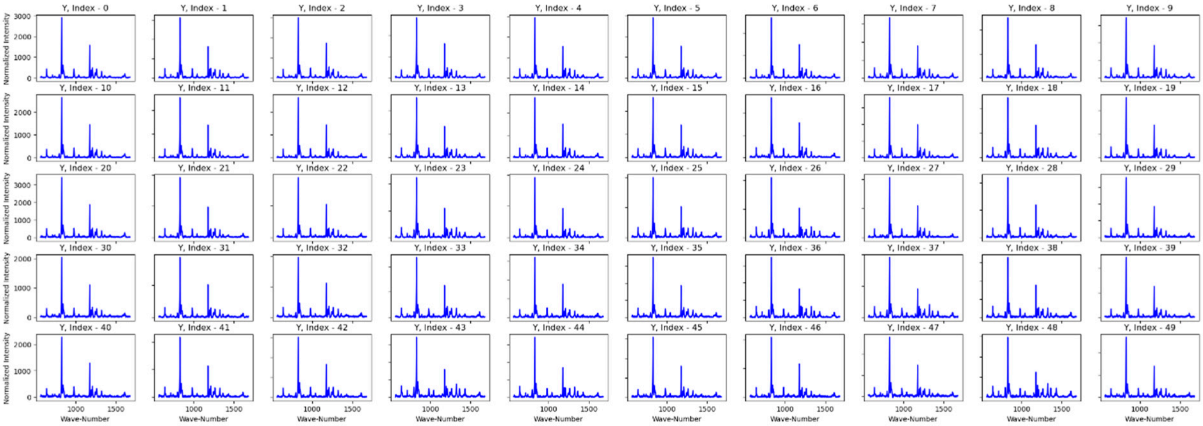

S3. SEM Images of SERS Substrate

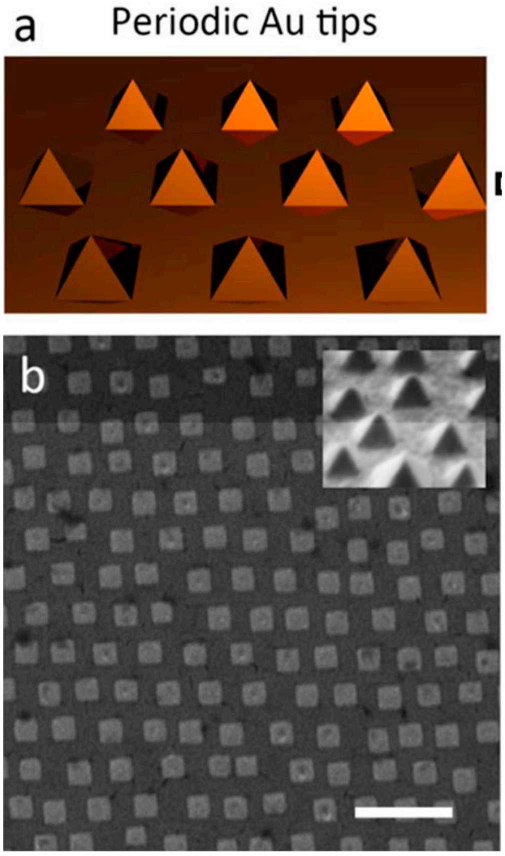

(a) 3D model of our Au-nanopyramid SERS Substrate
(b) SEM image of our SERS Substrate

ACS Anal. Chem. 2015, 87, 20, 10255–10261

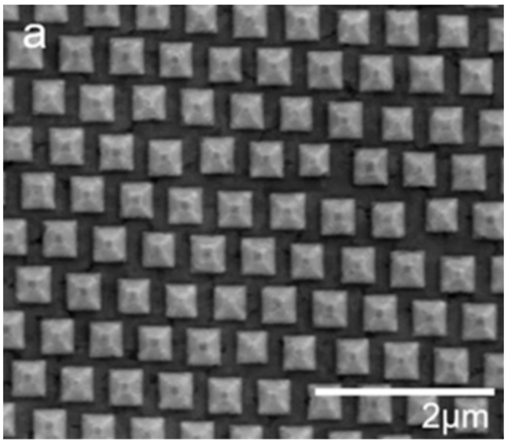

(a) SEM image of our SERS Substrate

ACS Appl. Nano Mater. 2022, 5, 9, 12506–12517

S4. Amino Acid Validation Data for the Trained Model

1. Alanine

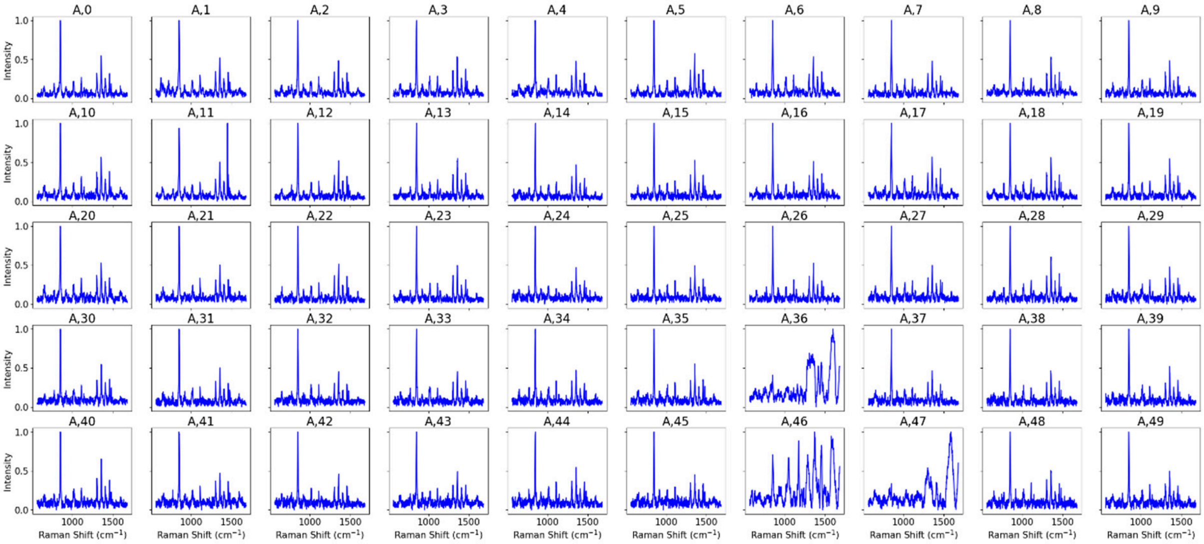

2. Cysteine

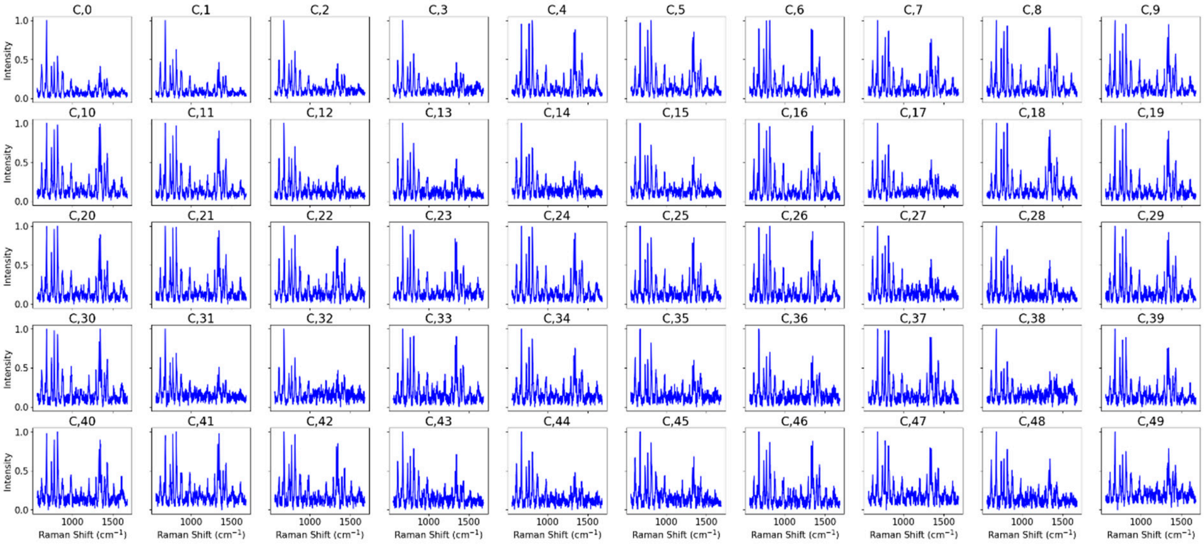

3. Aspartic Acid

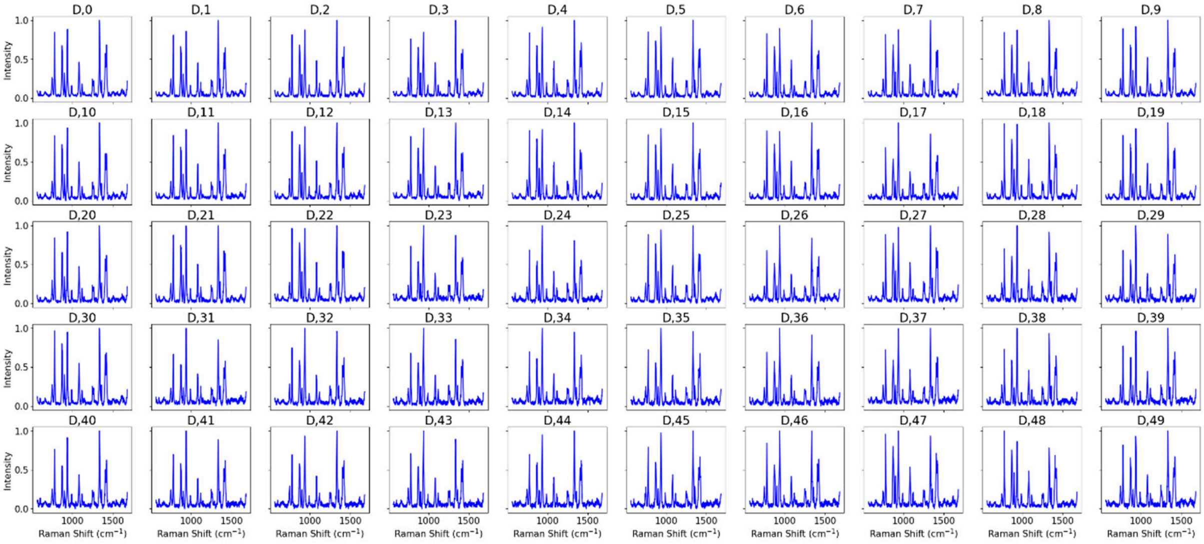

4. Glutamic Acid

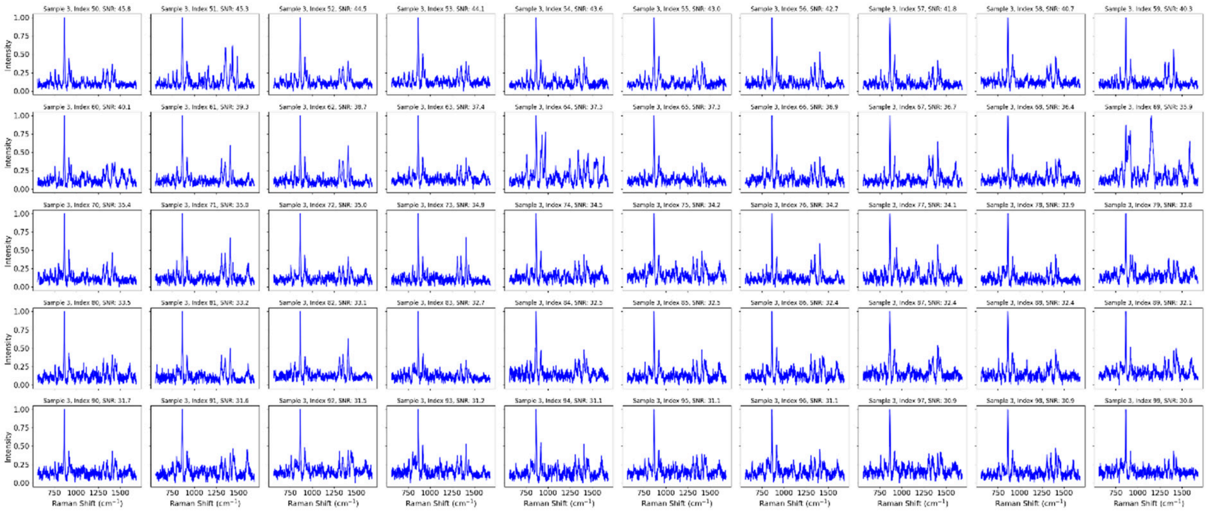

5. Phenylalanine

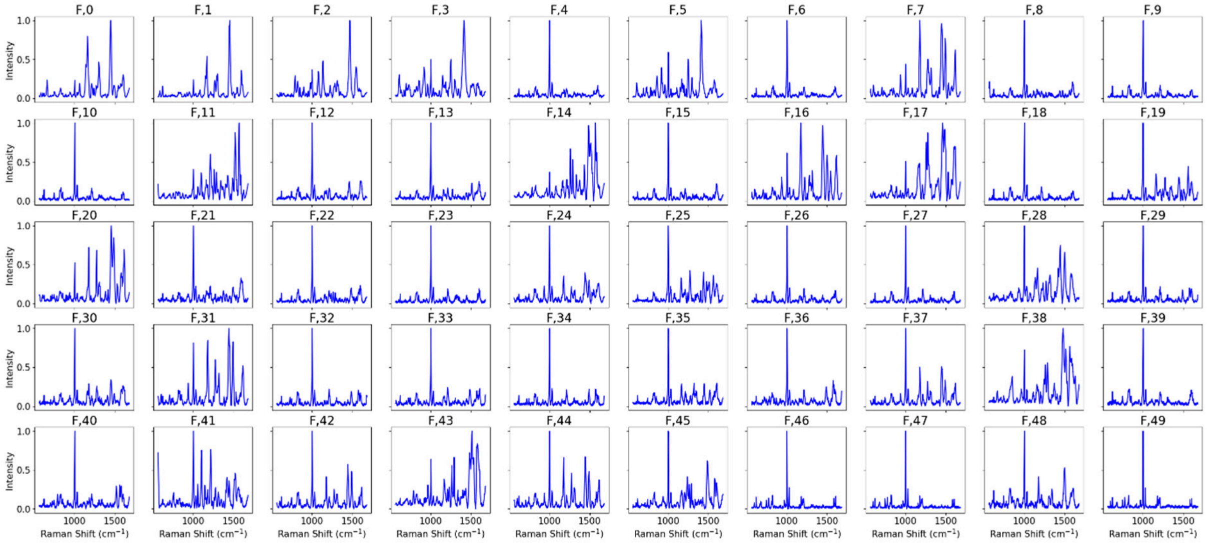

6. Glycine

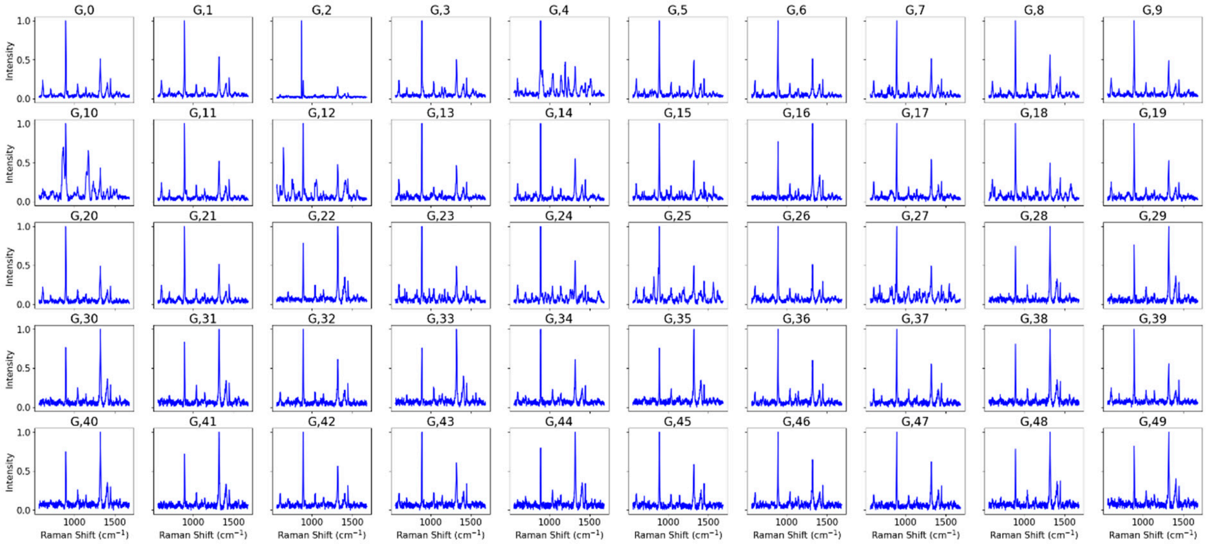

7. Histidine

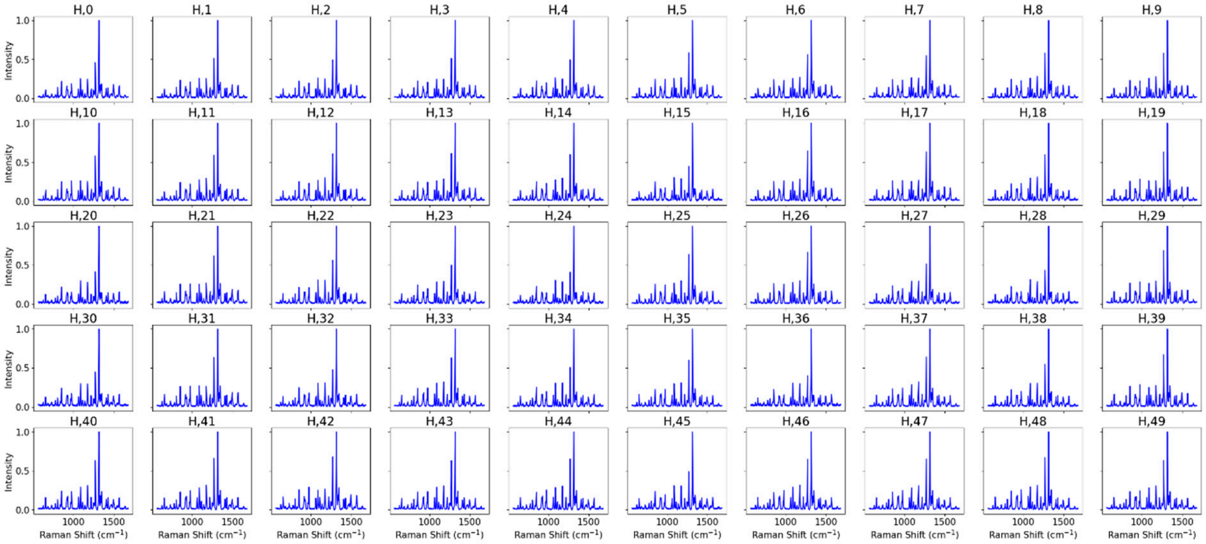

8. Isoleucine

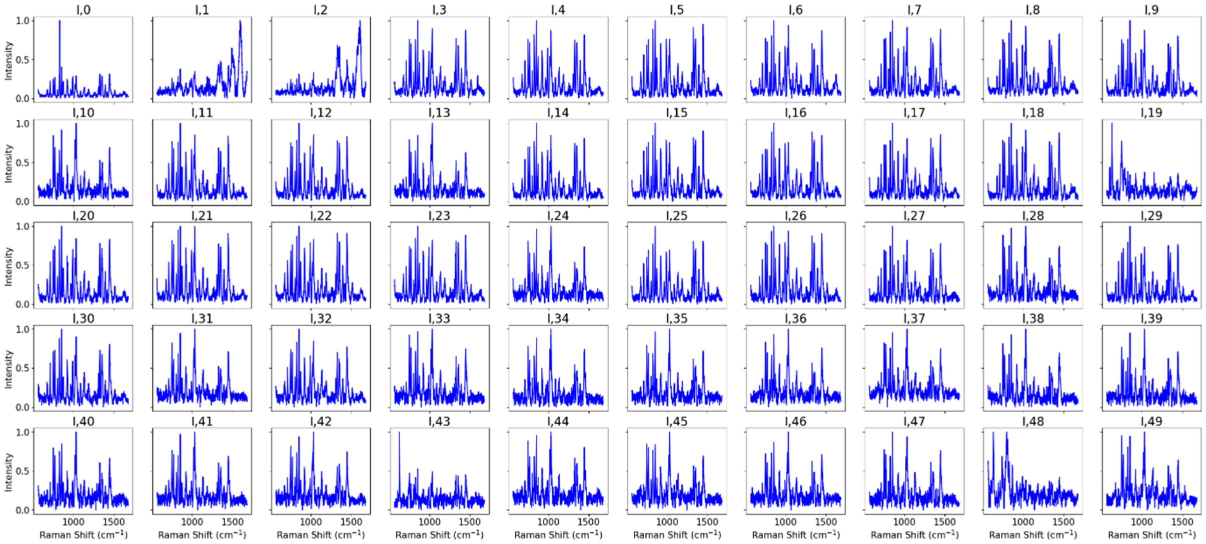

9. Lysine

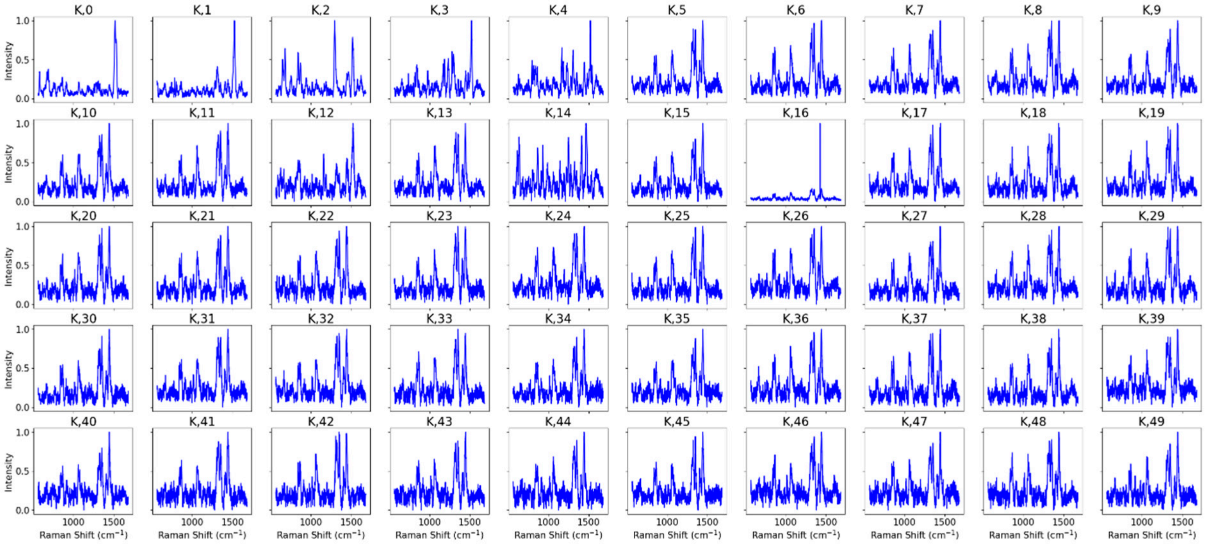

10. Leucine

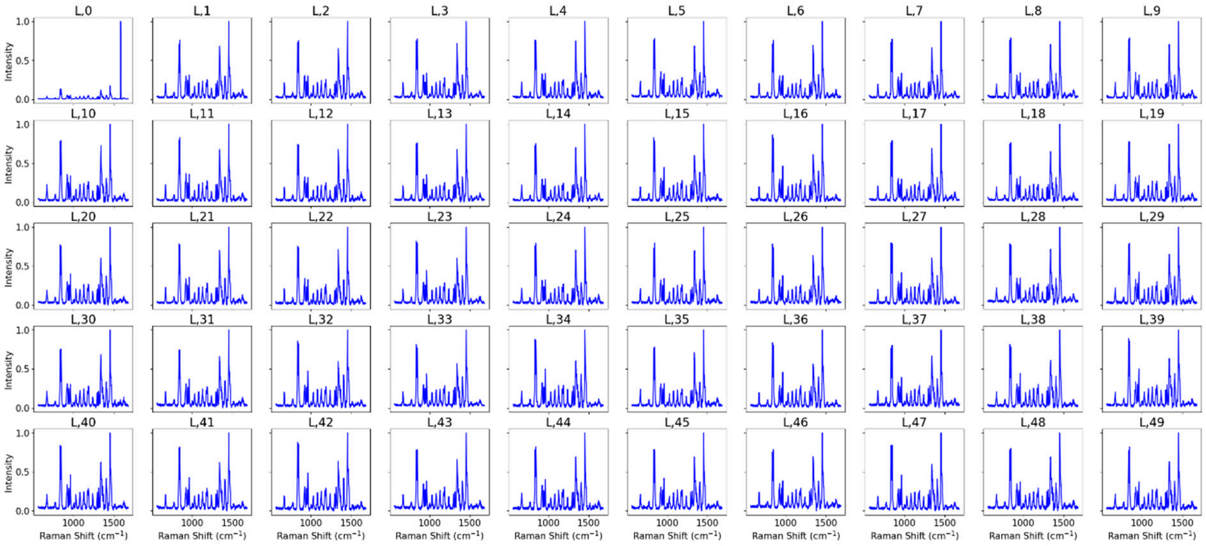

11. Methionine

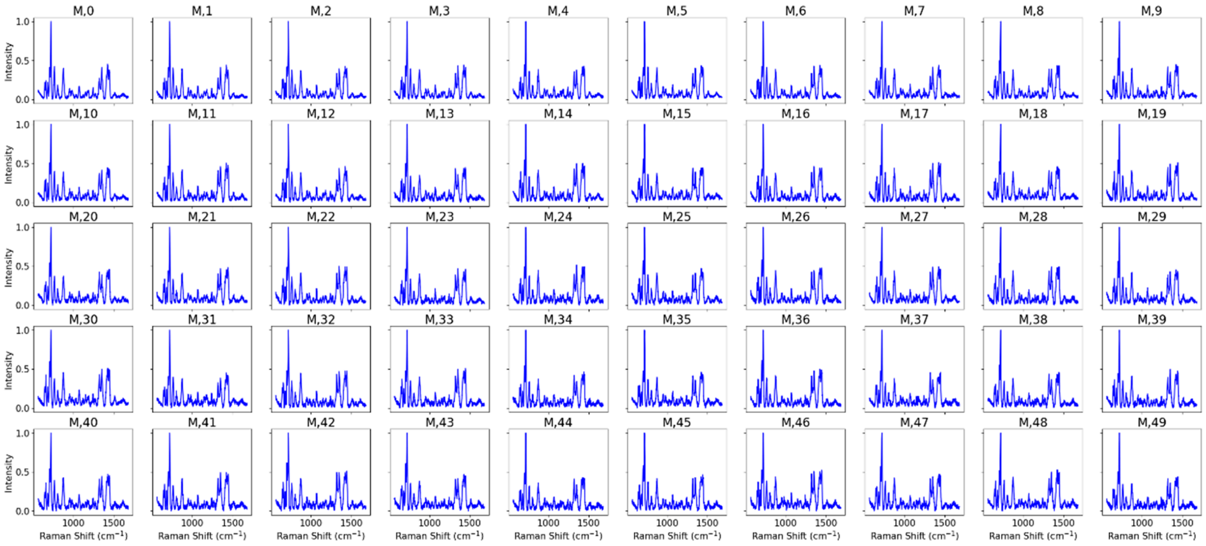

12. Asparigine

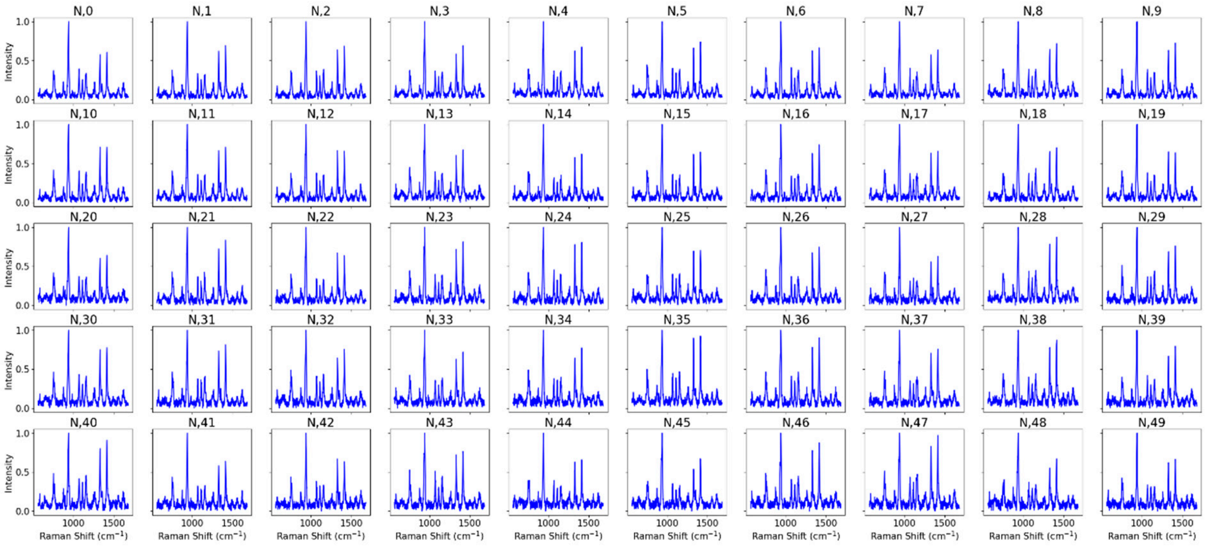

13. Proline

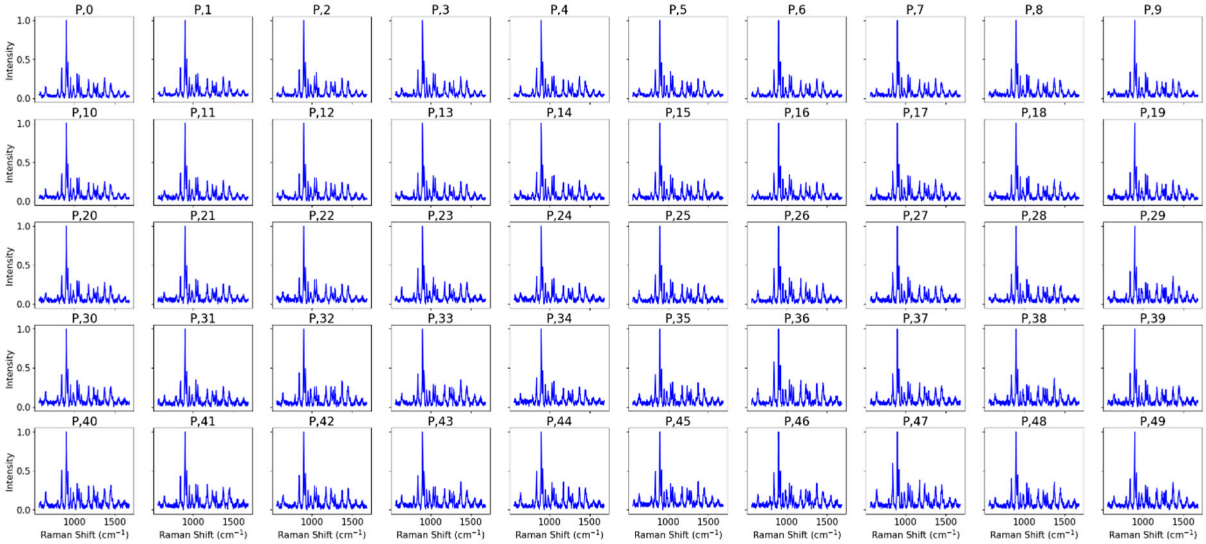

14. Glutamine

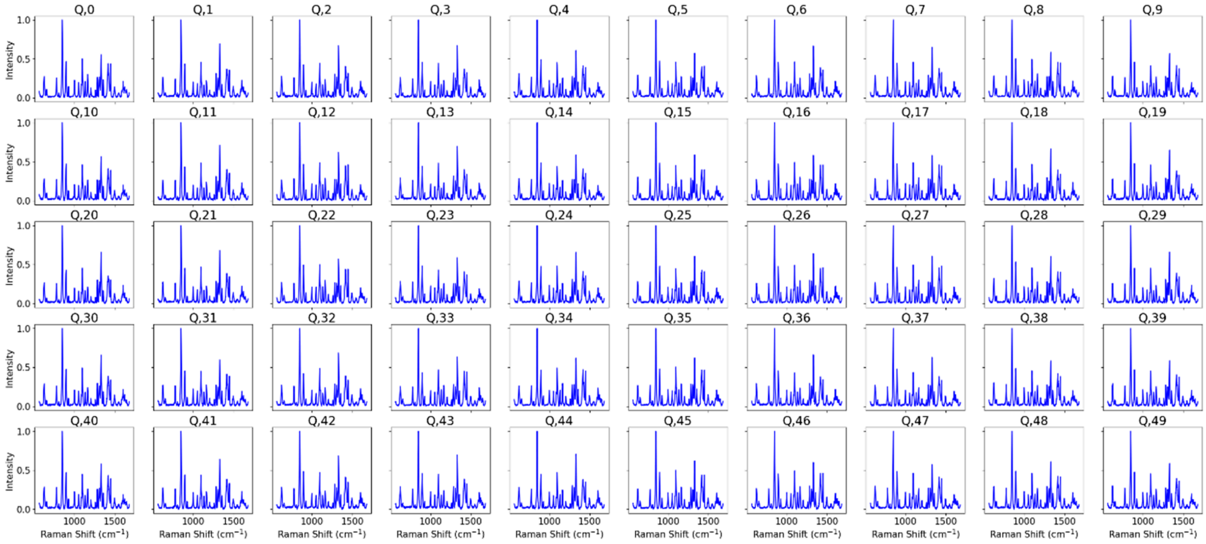

15. Arginine

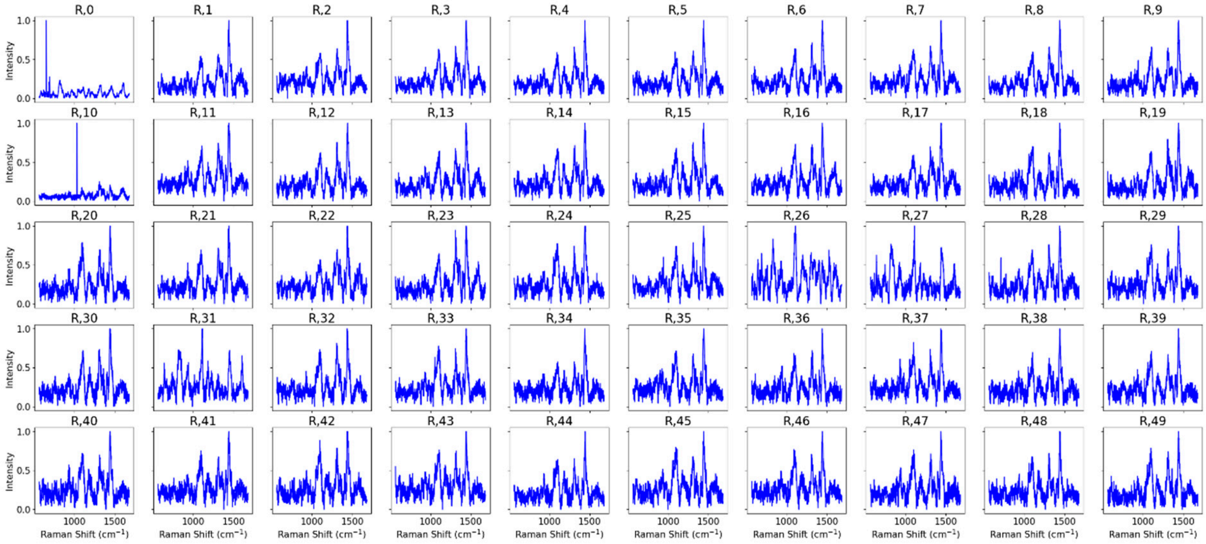

16. Serine

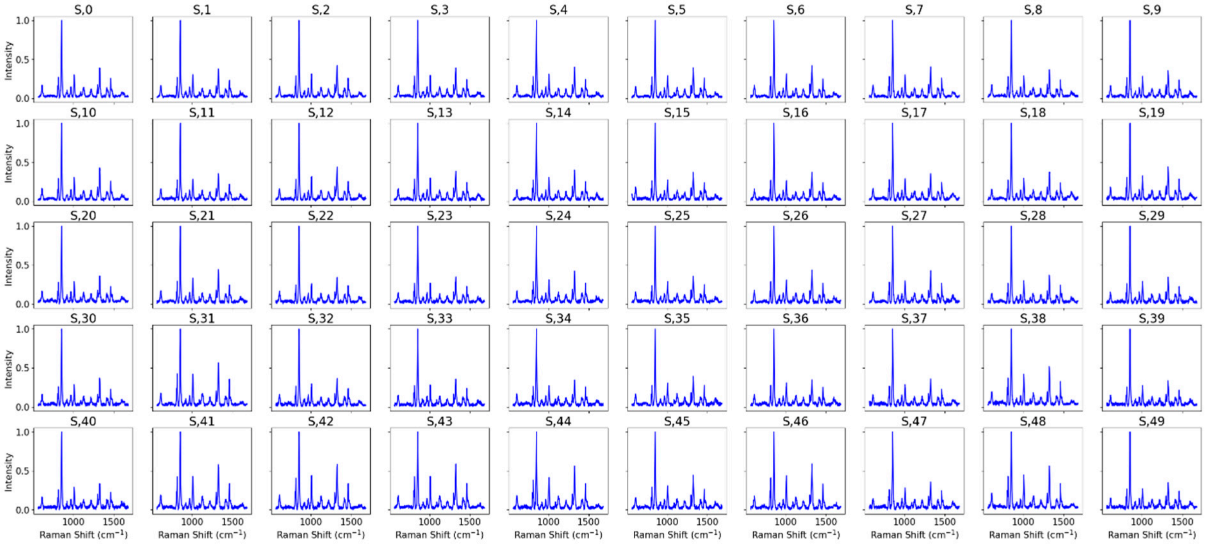

17. Threonine

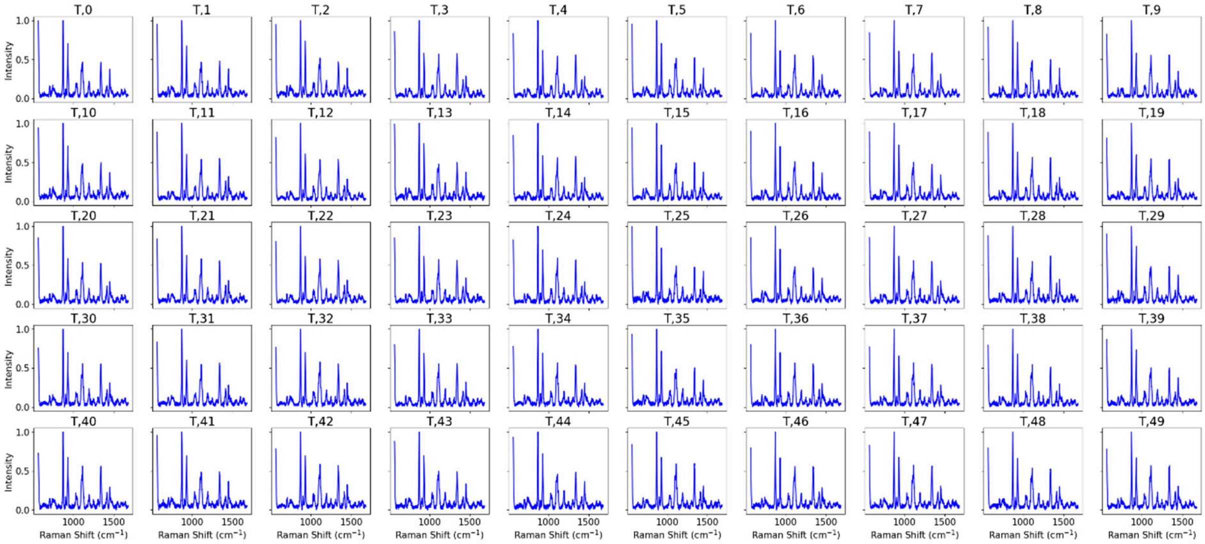

18. Valine

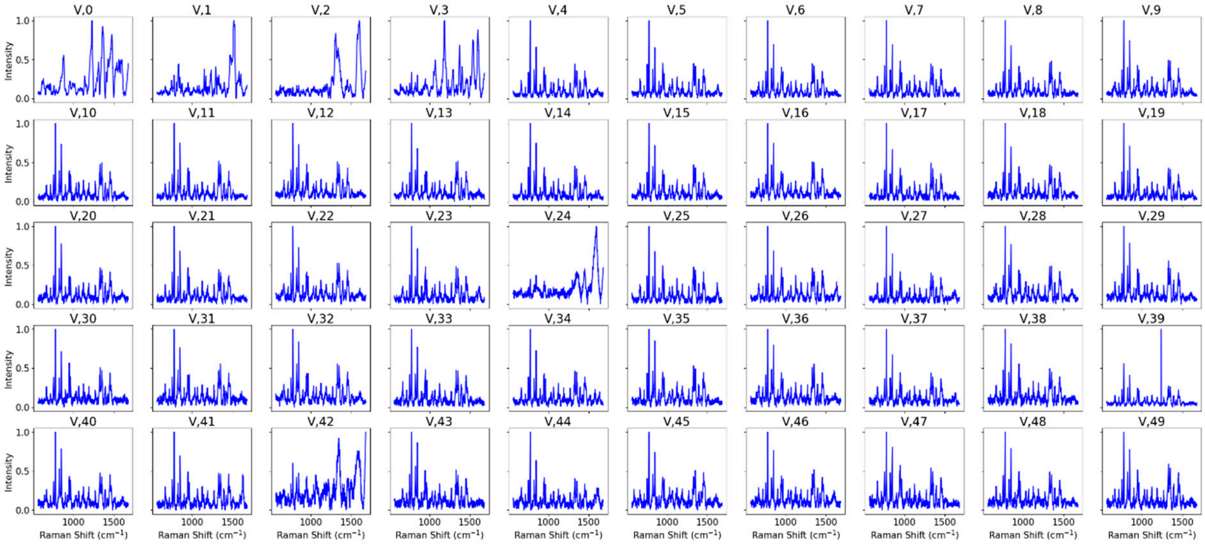

19. Tryptophan

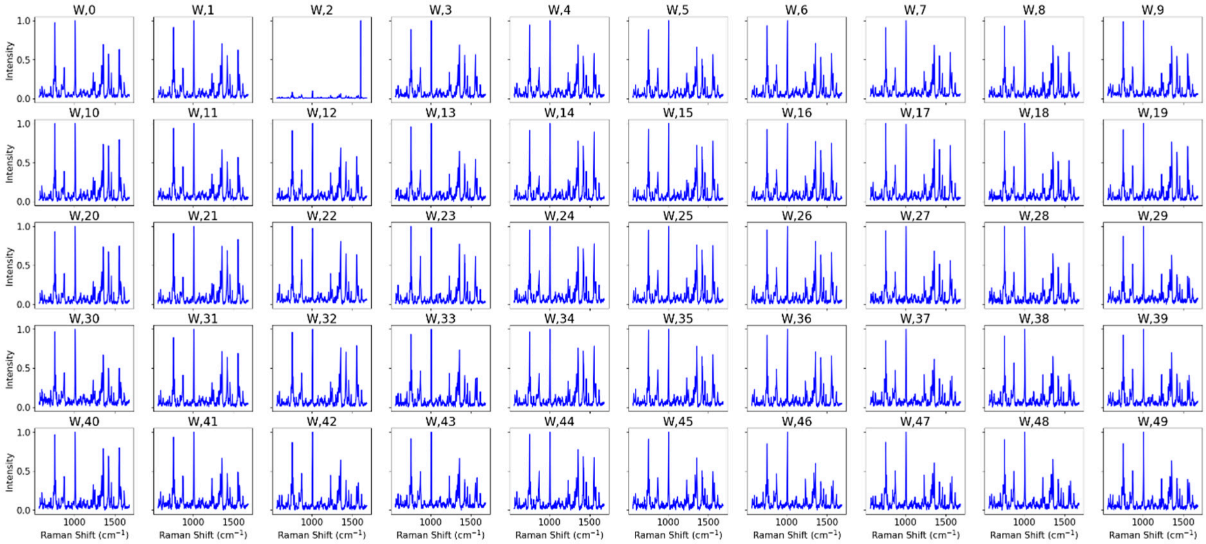

20. Tyrosine

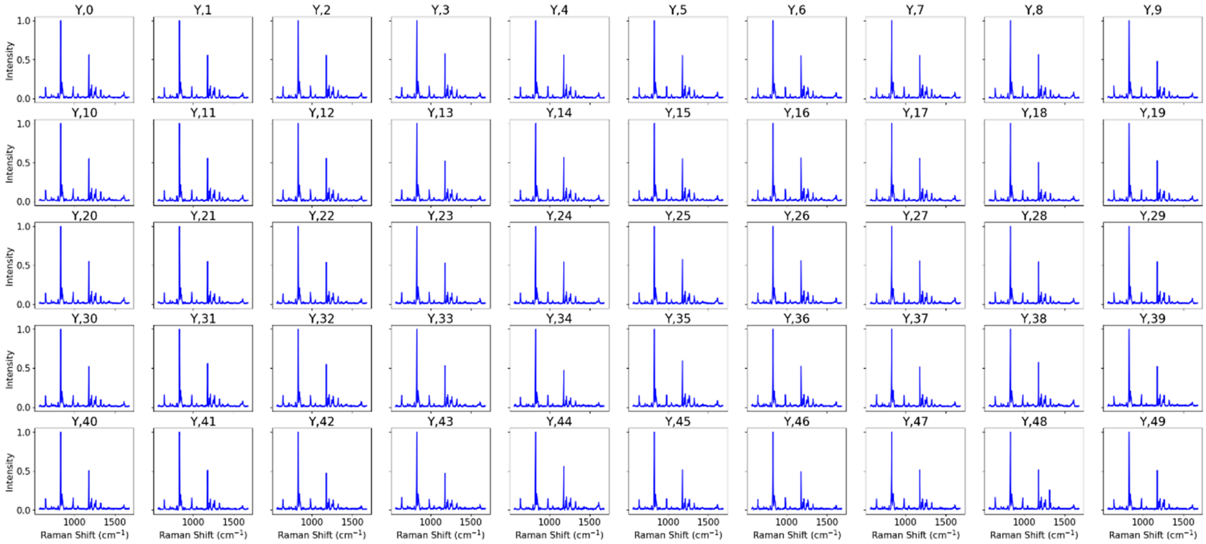

Supplement: Supplementary file 1 [file bioengineering-11-00482-s001.zip › bioengineering-2922529-supplementary.pdf]
